# Ultra-compact quintuple-band terahertz metamaterial biosensor for enhanced blood cancer diagnostics

**DOI:** 10.1371/journal.pone.0313874

**Published:** 2025-01-09

**Authors:** Musa N. Hamza, Mohammad Tariqul Islam, Sunil Lavadiya, Iftikhar ud Din, Bruno Sanches, Slawomir Koziel, Syeda Iffat Naqvi, Ali Farmani, Md. Shabiul Islam

**Affiliations:** 1 Department of Physics, College of Science, University of Raparin, Sulaymaniyah, Iraq; 2 Faculty of Engineering and Built Environment, Department of Electrical, Electronic and Systems Engineering, Universiti Kebangsaan Malaysia (UKM), Bangi, Selangor, Malaysia; 3 Department of Information and Communication Technology, Marwadi University, Rajkot, Gujarat, India; 4 Telecommunication Engineering Department, University of Engineering and Technology, Mardan, Pakistan; 5 Department of Electronic Systems Engineering, Escola Politécnica da Universidade de São Paulo, São Paulo, Brazil; 6 Engineering Optimization & Modeling Center, Reykjavik University, Reykjavik, Iceland; 7 Faculty of Electronics, Telecommunications and Informatics, Gdansk University of Technology, Gdansk, Poland; 8 Department of Telecommunication Engineering, University of Engineering & Technology Taxila, Taxila, Pakistan; 9 Department of Electronics Engineering, Lorestan University, Khorramabad, Iran; 10 Faculty of Engineering (FOE), Multimedia University (MMU), Cyberjaya, Selangor, Malaysia; Chulalongkorn University Faculty of Engineering, THAILAND

## Abstract

Cancer and its diverse variations pose one of the most significant threats to human health and well-being. One of the most aggressive forms is blood cancer, originating from bone marrow cells and disrupting the production of normal blood cells. The incidence of blood cancer is steadily increasing, driven by both genetic and environmental factors. Therefore, early detection is crucial as it enhances treatment outcomes and improves success rates. However, accurate diagnosis is challenging due to the inherent similarities between normal and cancerous cells. Although various techniques are available for blood cancer identification, high-frequency imaging techniques have recently shown promise, particularly for real-time monitoring. Notably, terahertz (THz) frequencies offer unique advantages for biomedical applications. This research proposes an innovative terahertz metamaterial-based biosensor for high-efficacy blood cancer detection. The proposed structure is ultra-compact and operates across five bands within the range of 0.6 to 1.2 THz. It is constructed using a polyethylene terephthalate (PET) dielectric layer and two aluminum (Al) layers, with the top layer serving as a base for the THz-range resonator. Careful design, architectural arrangement, and optimization of the geometry parameters allow for achieving nearly perfect absorption rates (>95%) across all operating bands. The properties of the proposed sensor are extensively evaluated through full-wave electromagnetic (EM) analysis, which includes assessing the refractive index and the distribution of the electric field at individual working frequencies. The suitability for blood cancer diagnosis has been validated by integrating the sensor into a microwave imaging (MWI) system and conducting comprehensive simulation studies. These studies underscore the device’s capability to detect abnormalities, particularly in distinguishing between healthy and cancerous cells. Benchmarking against state-of-the-art biosensors in recent literature indicates that the proposed sensor is highly competitive in terms of major performance indicators while maintaining a compact size.

## 1. Introduction

Cancer stands as one of the most intimidating diseases encountered by humans. By 2030, it is estimated that nearly 17 million people will die from cancer-related health issues, and also there will be 26 million new diagnosis [1]. Among the various types of cancers, blood cancer is particularly aggressive. Originating from bone marrow cells, blood cancer leads to the development and proliferation of abnormal blood cells, thus disturbing the production of normal blood cells [2].The occurrence of blood cancer is increasing every year due to genetic and environmental factors. The detection for older patients remains bleak in the face of advances in dose-intensification techniques that have shown promising results in younger patients [3]. Efforts to address cancer incidence and mortality include not only advancements in treatment methods but also initiatives focused on prevention, early detection, and improving access to healthcare services. Early detection is important if treatment outcomes are to be successful. However, distinguishing normal from cancerous cells is hampered by their similarities. For accurate diagnosis, it is required that bone marrow samples should be examined, which not only takes plenty of time but also relies heavily on the expertise of the pathologist. Despite their significant role, the probability of human error still remains with the pathologists. Hence, an early accurate diagnosis is important for increasing the recovery rates and reducing the blood cancer cases globally [4].

Until now, various techniques have been employed for blood cancer identification, including morphological examination with an electronic or optical microscope, DNA sequencing, fluorescence in situ hybridization (FISH), polymerase chain reactions (PCR), flow cytometry (FC), and immunohistochemistry [5,6]. However, these techniques suffers from limitations such as high costs, lack of specificity, lengthy operation times, and the need for experts [7]. Consequently, correctly recognizing malignant cells remains challenging. Recently, the evaluation with a bio-sensing interface seems an attractive alternative for detection of malignancy. Biosensors are indeed highly versatile tools with a wide range of applications in clinical diagnostics, industrial applications, environmental monitoring, and the food industry [8–12]. However, to be effective, biosensors need to provide precise measurements and be highly sensitive to detect even low concentrations of the target analyte. This is crucial for early diagnosis in clinical settings and accurate monitoring in other applications. Also, the response of the biosensor should be linear over a range of concentrations, allowing for straightforward interpretation of the data and reliable quantification. Moreover, for clinical trials and other applications where space and ease of use are critical, biosensors should be small and minimally invasive. This helps in reducing discomfort and potential damage to biological tissues. In addition, the sensor should also perform real-time analysis by providing immediate feedback. The ability to perform real-time analysis is essential for timely decision-making, in a medical context [13]. Biosensors come in various types, each suited to specific applications and offering unique advantages including electrochemical, amperometric, potentiometric, thermometric, and optical biosensors [14–18]. Optical bio-sensors are particularly valued for their high sensitivity, portability, label-free sample detection, low cost, and rapid sample preparation [19,20]. Optical sensors have various types such as photonic crystals, optical fibers, metamaterials (MMs), plasmon-induced transparency [21–24] etc.

Recently, Metamaterials (MMs) based sensors have gained great attraction due to their unique properties and versatility. MMs are artificially engineered electromagnetic materials designed to have properties not typically found in nature. They are characterized by unique physical properties that arise from their structure rather than their composition such as negative refractive index, negative permeability, and super absorption [25–27] etc. These unique properties make metamaterials highly versatile for a range of applications, including advanced optical devices, cloaking technology, improved telecommunications, enhancement of antennas for early cancer detection and sensing [28–33]. Metamaterial sensors are highly sensitive to their surroundings due to their unique ability to confine electric and magnetic fields on their surfaces. Tiny changes in the environment around the metamaterial sensor can lead to significant variations in its electromagnetic response, such as changes in optical absorption and resonance frequency [34]. This sensitivity makes metamaterial sensors exceptionally effective for various sensing applications including Refractive Index sensing, DNA detection, cancer cell identification, and microfluidic applications [21,35–38]. Thus, the ability of metamaterial sensors to detect such small changes with high precision makes them valuable tools in medical diagnostics. Metamaterial (MM) biosensors are classified based on their operating frequency regimes into several groups of microwave [39], terahertz [40], and plasmonic [41], each with distinct characteristics and applications. As the operating frequency of the MM biosensor increases, the sensor’s dimensions become smaller. This miniaturization allows for the detection of samples with very small thicknesses [42], enhancing the sensor’s ability to detect minute changes and details in biological samples. By reducing the size of biosensors and their components, it becomes possible to enhance portability, usability, and integration, while also improving critical performance metrics like sensitivity and detection limits. This not only contributes to more accessible and frequent health monitoring but also helps achieve more accurate and timely diagnostics. Miniaturization allows for the development of complex biosensor systems that integrate various functionalities (such as sampling, sensing, and signal processing) within a single, lightweight platform. These platforms enable more rapid, automated, and parallel analysis, crucial for point-of-care diagnostics where fast, reliable results are needed. Moreover, it lowers production costs, as smaller devices consume fewer materials, and streamlines manufacturing processes [43]. As for the technical advantages, miniaturization tends to increase the signal-to-noise ratio of electronic and electrochemical biosensors [44]. This makes them more sensitive, improving their limit of detection, which is essential for identifying low concentrations of biomarkers or analytes early on. By reducing the response time, such sensors can deliver real-time monitoring or diagnostics, which is key for wearable biosensors meant to provide continuous health insights. Thus, miniaturization is not just about making biosensors smaller, it is about optimizing performance while increasing accessibility and affordability in healthcare monitoring.

The terahertz (THz) wave spectrum, which lies between microwave and infrared waves, offers unique advantages for biomedical applications. THz waves do not cause the ionization or damage to living tissues and the human body due to very low energy, thus ensuring the safety for medical imaging and diagnostics. Also, many biological macromolecules and polar molecules have vibrational and rotational energy levels within the THz region, thus, this resonance permits THz waves to interact specifically with these molecules, making it a powerful tool for characterizing biological tissues [45,46]. Hence, due to the unique interactions between THz waves and biological molecules, THz spectroscopy can be applied in various medical diagnostic applications. THz imaging can provide detailed images of biological tissues without the need for invasive procedures. This capability is beneficial for early detection and monitoring of diseases.

THz waves are strongly absorbed by water, which is a significant advantage in distinguishing cancerous tissue from normal tissue. Cancerous tissues typically have a higher water content than normal tissues. By analyzing the THz absorption spectrum, medical professionals can detect differences in water content and, therefore, differentiate between cancerous and healthy tissues. Therefore, the use of THz waves in medical diagnosis and tissue characterization is a growing field, offering a combination of safety, specificity, and non-invasive diagnostic capabilities [47–49].

Over the past two decades, THz time-domain spectroscopy (THz-TDS) technology has gained significant attention for its potential in detecting various types of cancerous tissues. Numerous studies have demonstrated the feasibility and effectiveness of THz technology over traditional methods in identifying cancerous tissues for skin, liver, breast, stomach, and colon [45,50–52]. Each type of cancer presents distinct characteristics in terms of THz absorption and refractive index, allowing for specific diagnostic applications. Also, THz imaging provides real-time feedback, enabling immediate visualization and assessment of tissue abnormalities during diagnostic procedures [53,54]. Overall, the promising results from studies using THz-TDS technology underscore its potential as a valuable tool in the early detection and characterization of cancerous tissues across various medical disciplines.

Hence, metamaterial based biosensors in THz spectrum meets the increasing request on detection and distinguishing different cancer cells in the human biological tissues. Lately, various research works reported metamaterial based sensors operating in the THz range for cancer detection [55–64]. The work in [57] described four MTM based biosensors for the detection of cancer affected cells including blood cancer, adrenal gland cancer, cervical cancer, breast cancer, and skin cancer. The proposed structures obtained highest sensitivities of 0.06667 THz/RIU, 0.06667 THz/RIU, 0.2 THz/RIU, and 0.20714 THz/RIU, with the Quality factor (Q factor) and Figure of merit (FOM) of 13.11 and 3.86 RIU^−1^ respectively. Another work [59], proposed a metamaterial based biosensor operating in the THz range which demonstrates an average sensitivity of 495 GHz/RIU (7.64 dB/RIU) and a quality factor of 82. This biosensor is proposed to detect and distinguish between various healthy and cancerous cells including the Jukrat cells. Similarly, in [61] a surface plasmon resonance biosensor is proposed for accurate detection of blood cancer cells with sensitivity of 232.25 deg/RIU. In [62], a triple-band MTM based biosensor in the THz range is suggested for different cancer cells identification. The proposed sensor can differentiate between different cancer cell types such as skin cancer, blood cancer, and Breast cancer. This sensor achieved sensitivity of 2.050 THz/RIU with Q-factor of 55.34. Also in [65], a double split ring resonator based sensor using graphene material backed by MgF2 substrate is proposed for blood cancer detection. This sensor exhibited overall sensitivity of 3571 nm/RIU, FOM value of 714 RIU^-1^, and Q-factor with value 322. Another THz metamaterial based sensor is reported in [66]for early detection for basal, breast, Jurkat and Cervical cancerous cells. The reported sensor realized an average sensitivity of 556.325 GHz/RIU with Q-factor of 13.2. In the same way, a graphene-based metamaterial biosensor is presented for early cancer detection in [67]. This three circular graphene split ring resonators based sensor attains a maximum sensitivity of 3.880 THz/RIU, a Q-factor of 8.948, and a figure-of-merit of 8.146 RUI^-1^ enabling the device to differentiate between various cancer cell types, including skin, blood, cervical, adrenal gland, and breast cancer. Likewise in [68] a THz-biomedical sensor is demonstrated where peak sensitivity of 4.70, 5.50, 5.67, 4.50, 4.78, and 5.05 THz/RIU is obtained for blood, basal, cervical, jurkat, breast, and MCF-7 cancers, respectively, with figure of merits of 16.26, 19.16, 19.68, 12.85, 16.26, and 17.12 RIU^-1^. Furthermore, numerous biosensors have been designed for the early detection of various cancers and other biomedical applications. Recent studies have highlighted the development of metamaterial-based sensors in the terahertz (THz) frequency range for cancer cell detection [38,56–58,60,63,64, 69–76], alongside sensors targeting a range of bio-detection applications [35,77–92]. A significant portion of these cancer-detecting biosensors utilize graphene as the primary material [57,58,63,69]. Additionally, several designs incorporating ring-based resonator structures [38,56,69,72,74] have been proposed as effective approaches for THz metamaterial sensors in cancer cell detection.

The efficiency of terahertz (THz) band-operated biosensors is heavily dependent on microwave wire interference techniques. These biosensors are very sensitive to electromagnetic interference (EMI) from internal components and surroundings. Unwanted noise and signal interference may significantly reduce the accuracy of biosensor readings, making it difficult to detect biomolecules such as proteins, DNA, or other analytes at low concentrations. Microwave interference may significantly reduce cross-talk and isolate sensitive components from external noise by careful shielding. This allows for more consistent and exact readings, which is useful when working with complex biological materials.

Split-ring resonators, interdigital capacitors, or meander lines may concentrate the electromagnetic field in specific places, improving the interaction between THz waves and biological samples. This greater field confinement boosts the biosensor’s sensitivity, enhancing its capacity to detect minute changes in the biological environment. These improvements in sensitivity and resolution are crucial for applications like environmental monitoring and medical diagnostics, where precision is critical. Combining resonant structures with microwave wire interference techniques reduces noise and improves the biosensor’s overall performance, ensuring more consistent nanoscale detection of small biomolecular changes.

As, the tendency towards minimalism, compactness, and simplicity is particularly predominant in modern technology design, therefore, this work proposed an ultra-compact metamaterial based biosensor in the THz range with quintuple-band operation for blood cancer diagnostic. Cancer is a major threat to human health, with blood cancer being particularly aggressive. Originating from bone marrow, it disrupts normal blood cell production. Early detection is critical for better treatment outcomes. However, accurate diagnosis is challenging due to similarities between normal and cancerous cells. High-frequency imaging, especially terahertz (THz) frequencies, shows promise. This study introduces a compact terahertz metamaterial-based biosensor for blood cancer detection. Operating in the 0.6 to 1.2 THz range with >95% absorption rates, it uses a PET dielectric layer and aluminum layers. Full-wave EM analysis validates its effectiveness, distinguishing between healthy and cancerous cells, and proving competitive with current biosensors.

## 2. Structural design and incident field orientations

**Fig 1:**
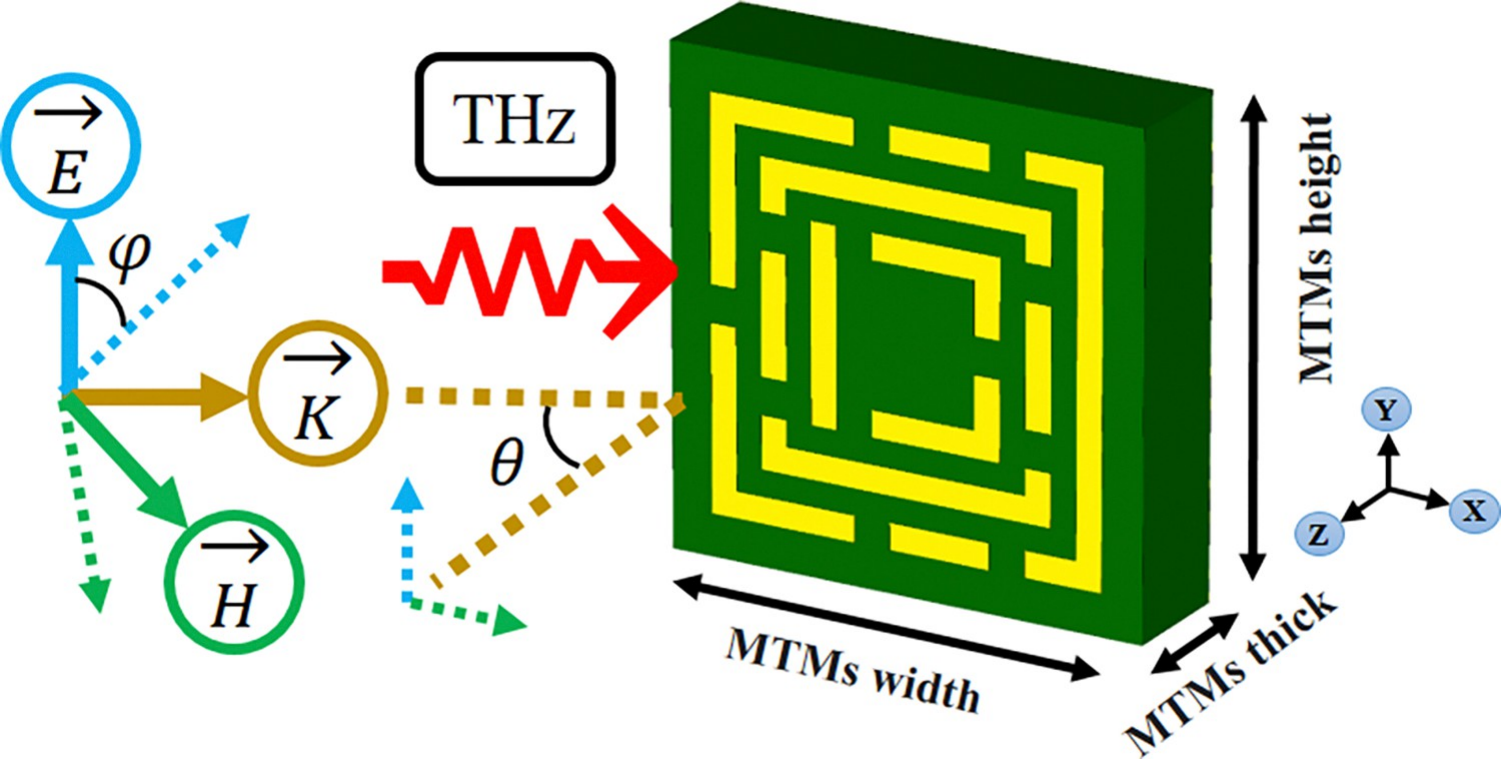
Diagram of the structural design and incident field directions for a perfect absorber.

The novel structural property is helpful for absorbing applied waves. The proposed MTM design makes absorption, reflection, and transmission possible, as shown in Fig 1. In order to reduce the amount of reflection, perfect absorbers absorb up most of the incoming energy, whereas reflected waves return to the MMT surface. While boosting absorption, the structure of the MMT decreases harmful interference. Transmission is reduced by using the energy of MMA waves. With the MMT structure, you may adjust the transmission characteristics more precisely by reducing or absorbing waves. The quantity of energy transmitted is reduced by perfect absorbers. The biosensor’s sensitivity, specificity, and selectivity should be carefully evaluated during the design process. For a precise blood cancer diagnosis, these characteristics are essential. Increasing the sensitivity and specificity of biosensors for this application might prove to be a formidable challenge. Improving the clinical value and providing valuable data for blood cancer diagnosis requires optimizing biosensor performance.

**Fig 2:**
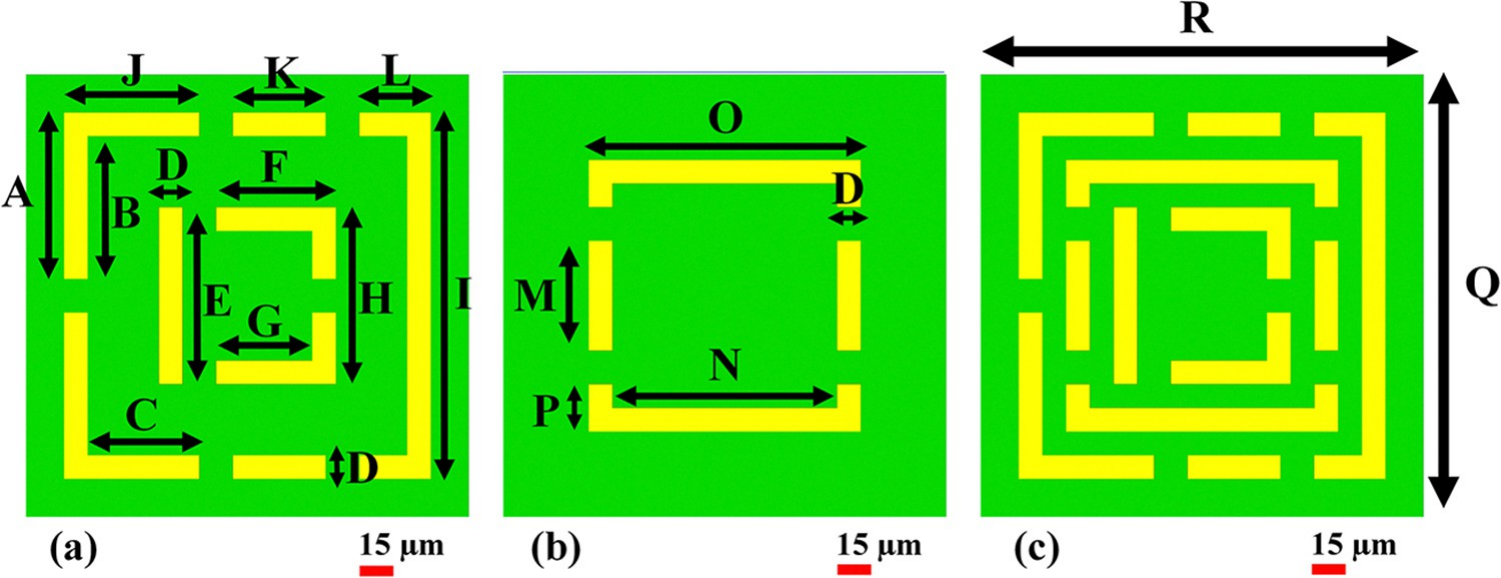
Recommended designs for a perfect absorber: (a) Model 1, (b) Model 2, and (c) Model 4 (proposed biosensor design).

Metamaterials play a pivotal role as optimal absorbers of Terahertz waves, offering significant impact for biosensors in the detection of blood cancer. Their unique design facilitate the minimization of wave reflection and the augmentation of absorption capabilities by exerting precise control over transmission. This becomes especially relevant in the context of biosensors designed for blood cancer detection, where the effective absorption of Terahertz waves is critical for obtaining accurate and insightful diagnostic data. The movement between metamaterials and Terahertz waves, reflected waves rebound from the metamaterials surface, while the design focuses on minimizing such reflections by absorbing a substantial portion of the incident energy. The careful structuring of the metamaterial also addresses the challenges of sensitivity and specificity, essential considerations in blood cancer diagnosis. As these challenges are opposed, optimizing biosensor performance emerges as a crucial objective. Despite inherent difficulties, the pursuit of enhanced sensitivity and specificity in biosensor design is indispensable for ensuring the clinical ability of blood cancer detection. Ultimately, this optimization not only elevates the diagnostic value but also adds to a more profound ability of blood cancer, development advancements in both clinical practice and research. The metamaterial perfect absorber depicted in Fig 2 integrates three models as shown in Fig 2A–2C to attain a five-band response within the terahertz spectrum. Consisting of three layers, each having conductive materials on both surfaces and a central dielectric substance, this arrangement deliberately addresses challenges arising from the overlap and interaction of magnetic and electric forces. The proposed design in Fig 2C aims at a significant enhancement in sensor capabilities. A summary of the models’ directional data is displayed in Table 1, providing an in-depth understanding of the details of the combined design and its potential for advanced sensing applications in the terahertz range. The absorber design employed sophisticated tools, utilizing the CST microwave studio, a high-frequency electromagnetic solver, and numerical analysis, especially the finite integration method. This approach facilitated a detailed examination of metamaterial (MTM) characteristics, exploring how MTM geometry function through various frequency spectrum ranges and boundary conditions. The three-layered structure of the absorber featured aluminum layers on top and bottom, ensuring robustness, while a polyethylene terephthalate dielectric spacer in the middle enhanced simulation efficiency. This approach enabled researchers to examine unit cells, open space, periodic arrays, as well as ideal electric and magnetic conductors, contributing valuable insights to metamaterial research.

**Table 1:** A complete list of the variables that have been adjusted for the recommended sensor.

Parameter	Value (μm)	Parameter	Value (μm)
A	74.524	K	41.491
B	63.917	L	32.024
C	49.926	M	49.195
D	10.606	N	100.4
E	79.195	O	121.62
F	53.589	P	21.213
G	42.982	Q	198
H	79.195	R	198
I	164.04	PET thick	50
J	60.533	Aluminum (Al) thick	0.2

The proposed terahertz metamaterial-based biosensor offers several notable advantages over existing biosensors, including cost-effectiveness, speed, ease of manufacturing, and compatibility with existing diagnostic systems. The sensor’s use of readily available and inexpensive materials, such as polyethylene terephthalate (PET) and aluminum (Al), can significantly reduce both initial and operational costs. Moreover, the sensor’s quintuple-band design enables rapid and efficient blood cancer detection by simultaneously analyzing multiple frequency bands, facilitating real-time monitoring. The sensor’s simple design and use of standard manufacturing techniques may further streamline the production process and potentially enable mass production, making it more accessible to a wider range of healthcare providers. Additionally, the sensor’s integration with microwave imaging (MWI) systems demonstrates its compatibility with existing diagnostic technologies, potentially reducing the need for significant infrastructure changes, while the proposed terahertz metamaterial-based biosensor shows promising advantages in terms of cost, speed, ease of manufacturing, and compatibility with existing systems, further research and development are needed to fully understand its limitations and ensure its optimal performance in real-world clinical settings.

The absorber included a 50 μm thick polyethylene terephthalate (PET) dielectric spacer, sandwiched between 0.2 μm thick aluminum (Al) layers on both sides. The impedance of the upper aluminum layer was adjusted to match that of the incident medium, facilitating optimal penetration and propagation of power within the polyethylene terephthalate. This ensured an efficient interaction with incoming electromagnetic (EM) waves. In contrast, the bottom aluminum layer, featuring zero impedance, served as an obstacle, obstructing incident waves in accordance with transmission-line theory (TLT). The primary objective of this design was to capture moving waves through substantial electrical or magnetic losses, thereby preventing their transmission via the bottom metallic layer.

The proposed biosensor’s exceptional absorption performance is a result of the synergistic interaction between its key components: the PET dielectric layer, the Al layers, and the MTM cell.

The PET dielectric layer, known for its low permittivity and small loss tangent, minimizes energy dissipation and maintains signal integrity in the terahertz range. This ensures high sensitivity in biomedical imaging, especially when detecting subtle variations between cancerous and healthy cells. Aluminum layers, chosen for their high conductivity and cost-efficiency, serve as a reflector and resonator structure. The high absorption rates (>95%) enhance the sensor’s ability to accurately detect biomarkers associated with blood cancer.

The PET dielectric layer acts as a dielectric medium, confining the electromagnetic field within the structure and enhancing the resonator’s interaction with incident terahertz (THz) waves. This strong interaction is essential for achieving high absorption across the operating bands. The aluminum layers, particularly the bottom layer, play a critical role in reflecting incident waves, trapping the energy within the resonator structure and preventing wave transmission. The high conductivity of aluminum enhances the formation of standing wave patterns, further contributing to near-perfect absorption at the resonance frequencies.

The MTM cell, engineered to resonate at specific THz frequencies, is the key to achieving the multi-band absorption observed in the biosensor. By carefully tuning the geometric parameters of the resonator, multiple resonance peaks within the target 0.6 to 1.2 THz range can be achieved. The metamaterial’s unique dispersive properties, optimized through full-wave electromagnetic simulations, allow for the simultaneous excitation of multiple modes, leading to high absorption at five distinct frequency bands, the combination of these components—PET dielectric layer, Al layers, and MTM cell—enables the biosensor to achieve exceptional absorption rates, making it a promising tool for blood cancer diagnostics.

The ultra-compact quintuple-band THz MTM biosensor, with its dimensions of 198×198 μm^2^, significantly enhances its practical utility for early blood cancer detection. Its compact design facilitates the integration of multiple sensors and sample preparation devices into a single, lightweight unit, promoting portability and enabling multiplexed, parallel, and automated analyses essential for point-of-care applications. Furthermore, this design reduces manufacturing costs by minimizing material usage and simplifying fabrication processes, thereby improving accessibility to advanced diagnostic technologies. The biosensor’s small size allows for seamless integration with electronic components, enhancing real-time monitoring capabilities and achieving higher sensitivity through an improved signal-to-noise ratio. Collectively, the compact nature of the biosensor positions it as a viable and effective solution for early blood cancer diagnostics, with ongoing research aimed at further optimizing its performance and accessibility.

## 3. Absorption properties and characteristics

**Fig 3:**
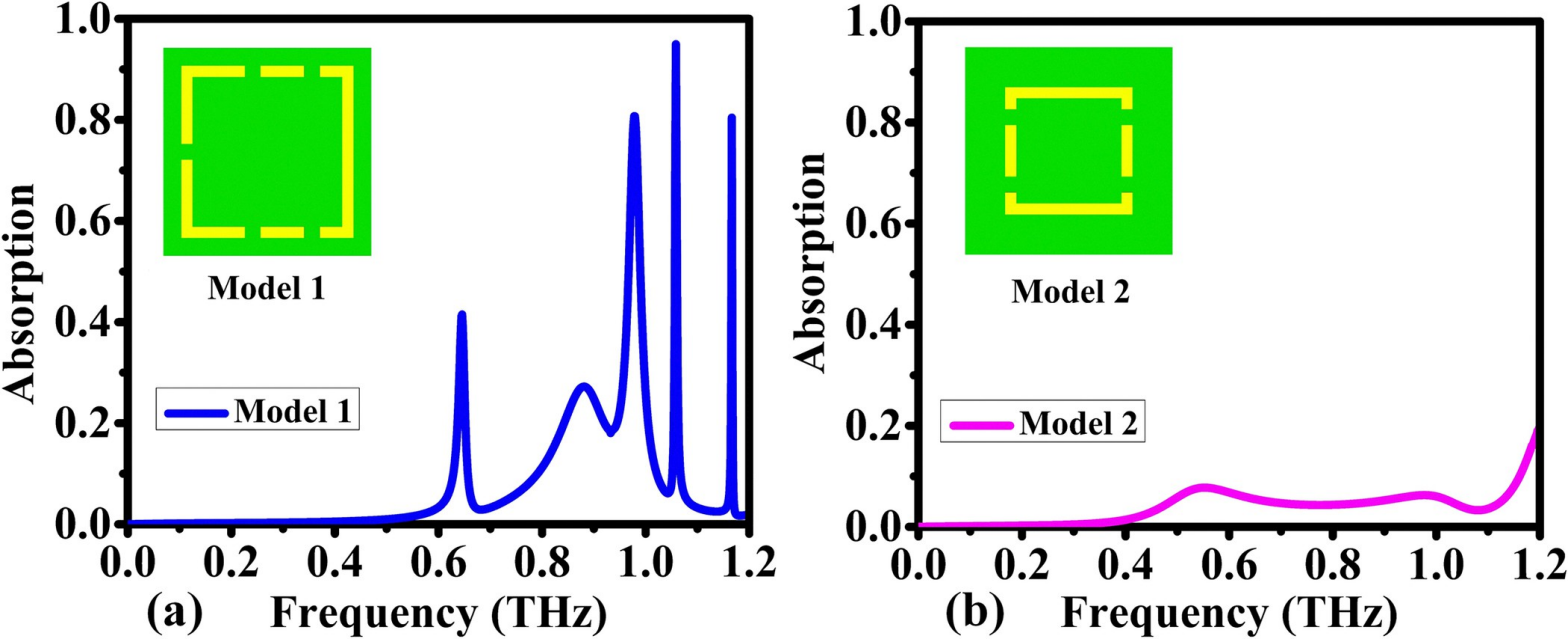
Comparison of the absorption characteristics for different designs: (a) Model 1, and (b) Model 2.

The initial configuration, as shown in Fig 3A, consists of five peaks functioning at frequencies of 0.6, 0.8, 1, 1.1, and 1.2 THz respectively. These peaks demonstrate absorption levels below 45% for the first two and above 80% for the subsequent three peaks. In contrast, using single split-ring resonators, the second configuration displays low absorption of less than 15%, ranging from 0.5 to 1.1 THz spectrum. Constraining the frequency to 1.2 THz aligns with current lab capabilities and promotes resonance between the first and second models when integrated.

**Fig 4:**
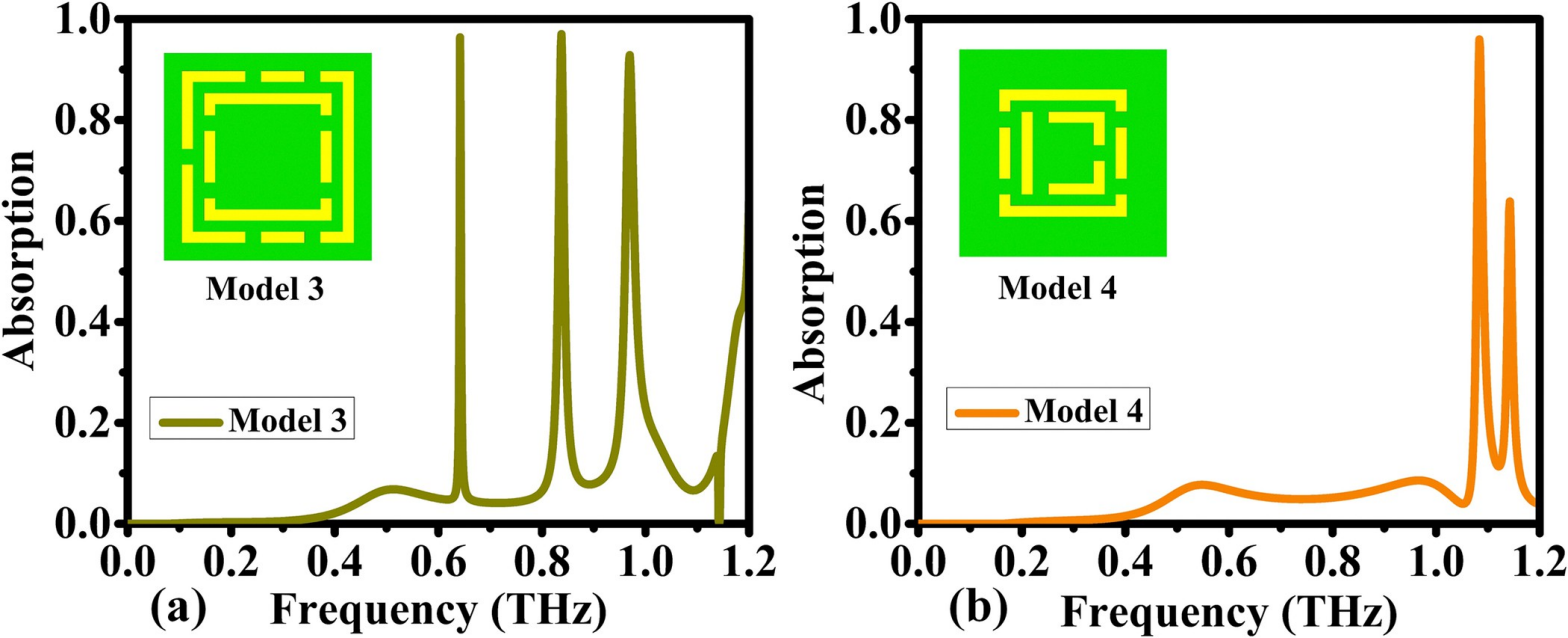
Comparison of absorption properties for various designs: (a) Model 3, and (b) Model 4.

In the domain of sensor technology, the capabilities of Model 3 and Model 4 are illustrated in Fig 4A and 4B. Model 3 operates adeptly at 0.6, 0.8, and 1 THz, boasting an impressive absorptivity exceeding 95%. Meanwhile, Model 4 operates at 1.1 and 1.2 THz, depicting absorptivity values of 98% and 63%. The high absorptivity of Model 3 across the specified terahertz frequencies positions it as a reliable sensor for signal interaction. On the other hand, Model 4, with its specific focus on 1.1 and 1.2 THz, demonstrates varying absorptivity levels, indicating distinct sensor capabilities within this higher frequency range.

**Fig 5:**
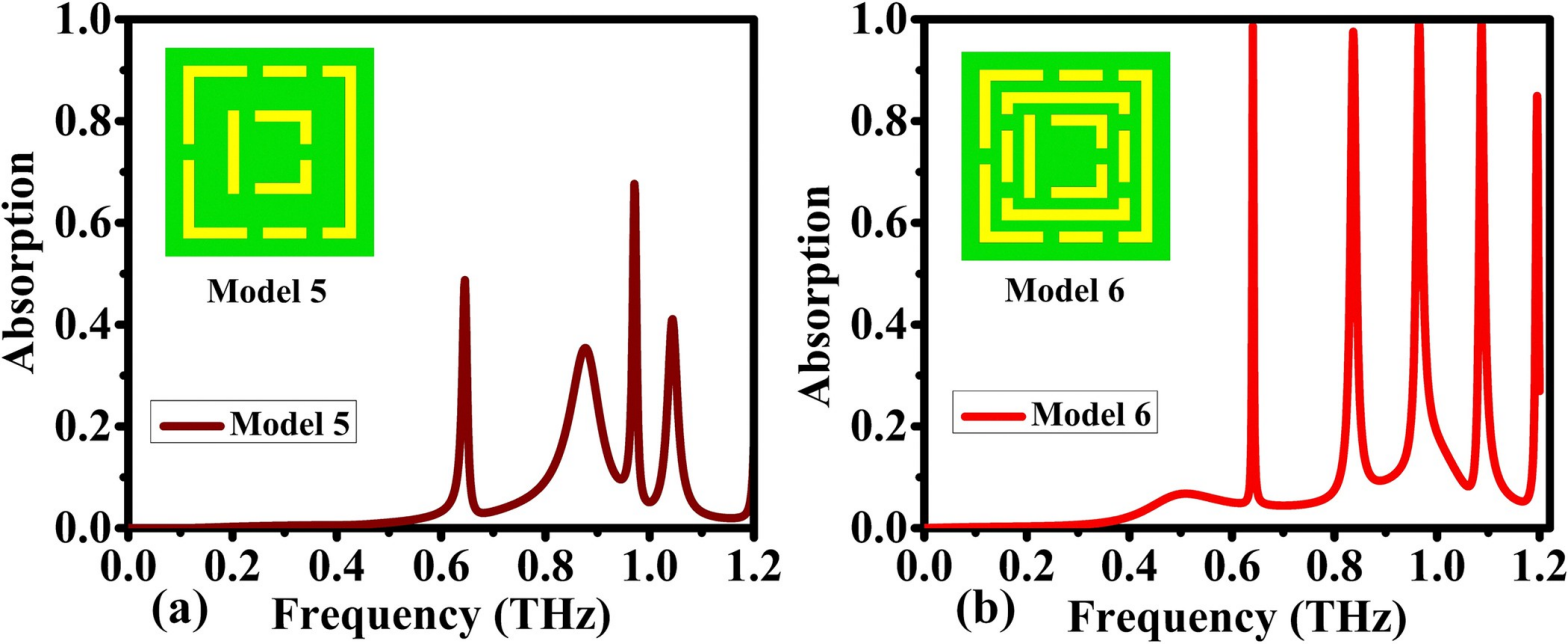
Comparison of absorption properties for various designs: (a) Model 5, and (b) Model 6.

Model 5 absorption capacities at 0.6 THz is 55%, 0.88 THz is 35%, and 1 THz is 70% respectively, as depicted in Fig 5A fall below 80%, indicating limitations for bio sensing applications due to insufficient sensitivity. Falling short of 80% absorption, is not considered a perfect absorber and thus unfit as a sensor independently. However, its value manifests when combined with other models. This interaction is highlighted in the final proposed model, demonstrating a notable improvement in absorption capacity. Together, these models create a more effective system, focusing on the collaborative nature required for optimal performance in the absorption domain. In contrast, the versatility of Model 6 as shown in Fig 5B takes center stage, as it operates across a spectrum ranging from 0.65 to 1.2 THz. Remarkably, the proposed model exhibits exceptional absorptivity peaks for the first four frequencies, surpassing an impressive 99% of absorptivity. This signifies a heightened sensitivity, suggesting a potential breakthrough in the early and accurate detection of blood cancer. Even for the fifth frequency at 1.2 THz, the absorptivity remains above 80%, highlighting the robustness of the model across a diverse set of terahertz frequencies. Model 6, with its broader operational range and consistently high absorptivity, emerges as a particularly promising way for advancing the precision and reliability of blood cancer diagnostics. This understanding lays the foundation for future research and development in the pursuit of more effective and versatile blood cancer detection models.

## 4. Electromagnetic response and field distribution analysis

This section provides an in-depth analysis of the electromagnetic behavior and field distribution characteristics of the metamaterial-based biosensor. It encompasses material and geometric optimization, investigates material attributes such as refractive index, and presents detailed visualizations of the E-field and H-field distributions. Furthermore, it examines surface current distributions, offering insights for enhancing the design and performance of the biosensor in detecting electromagnetic interactions.

### 4.1 Material and geometrical optimization

**Fig 6:**
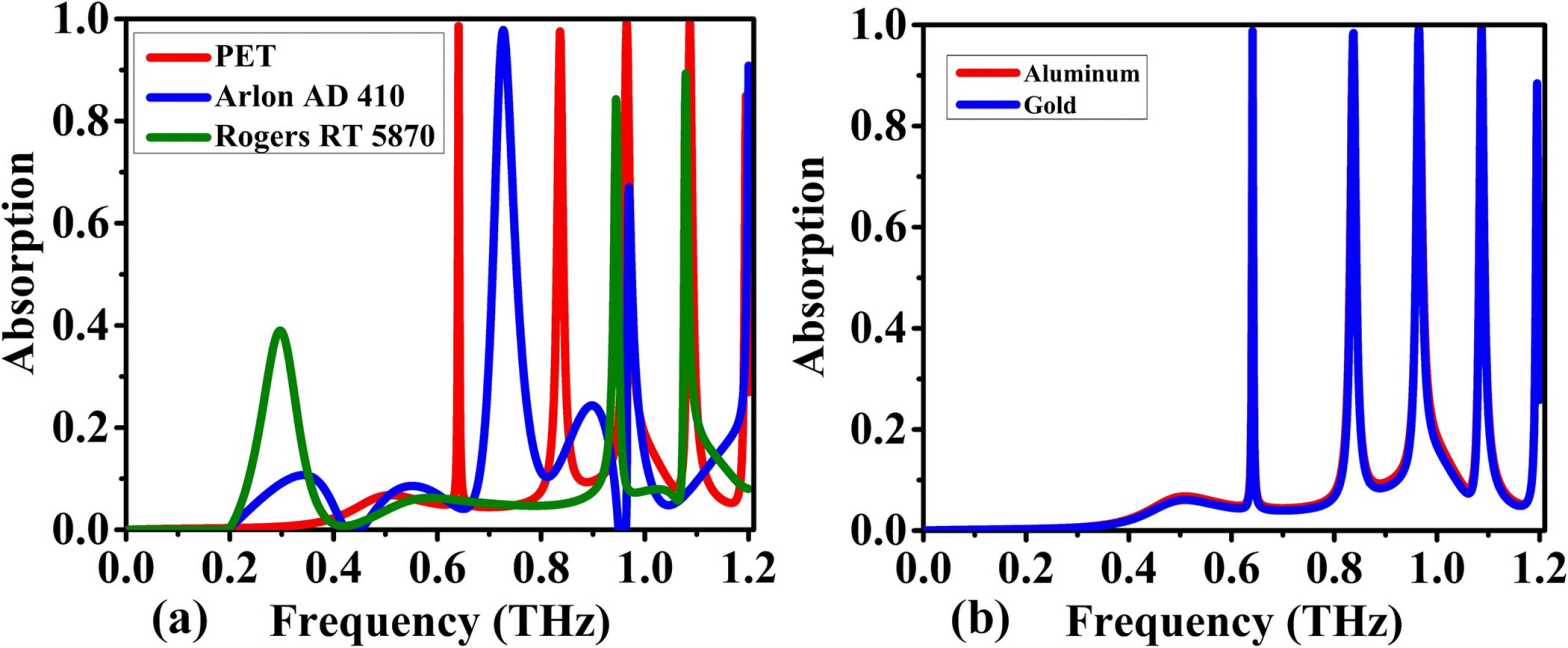
Absorption spectra for the proposed design under varying conditions: (a) substrate material, and (b) resonator material.

The selection of substrate materials in sensor design plays a pivotal role in the overall performance, particularly in terahertz frequency applications. In Fig 6A illustrates a comparative analysis of three substrates: PET (Polyethylene Terephthalate), Arlon AD 410, and Rogers RT 5870, focusing on their absorptivity and sensitivity within the 0 to 1.2 THz range. PET emerges as a notable contender, showcasing good absorptivity and sensitivity above 99% at first four frequencies and above 85% at 1.2 THz respectively. Its effectiveness in this context suggests its potential as a reliable substrate for sensors operating in the specified frequency range, providing a balance between absorption efficiency and sensitivity. On the other hand, Arlon AD 410 stands out for its impressive absorptivity of 99, 65 and 85% at 0.7, 0.9 and 1.2 THz, positioning it as a substrate with a strong capability to absorb terahertz radiation. This attribute is crucial for enhancing the sensitivity and overall performance of sensors, making Arlon AD 410 a promising choice for applications demanding heightened absorptive properties in the specified frequency range. In contrast, Rogers RT 5870, while not matching Arlon AD 410 in absorptivity, still offers favorable absorptivity of 40, 80 and 85% at 0.3,1 and 1.1THz respectively for sensor applications. The comparative analysis emphasizes the importance of balancing absorptivity and other material properties based on the specific requirements of the sensor system. It’s noteworthy that resonating materials, such as aluminum and gold as shown in Fig 6B, exhibit a uniform response in the 0 to 1.2 THz range. This consistency suggests that, in terms of resonance, both aluminum and gold can provide similar performance characteristics for sensors operating within this frequency spectrum. In conclusion, the sensitivity comparison of PET, Arlon AD 410, and Rogers RT 5870, along with insights into resonating materials, provides valuable considerations for sensor designers seeking optimal substrate materials for terahertz applications. The selection should align with the desired balance between absorptivity, sensitivity, and resonance characteristics based on the specific demands of the sensor system.

**Fig 7:**
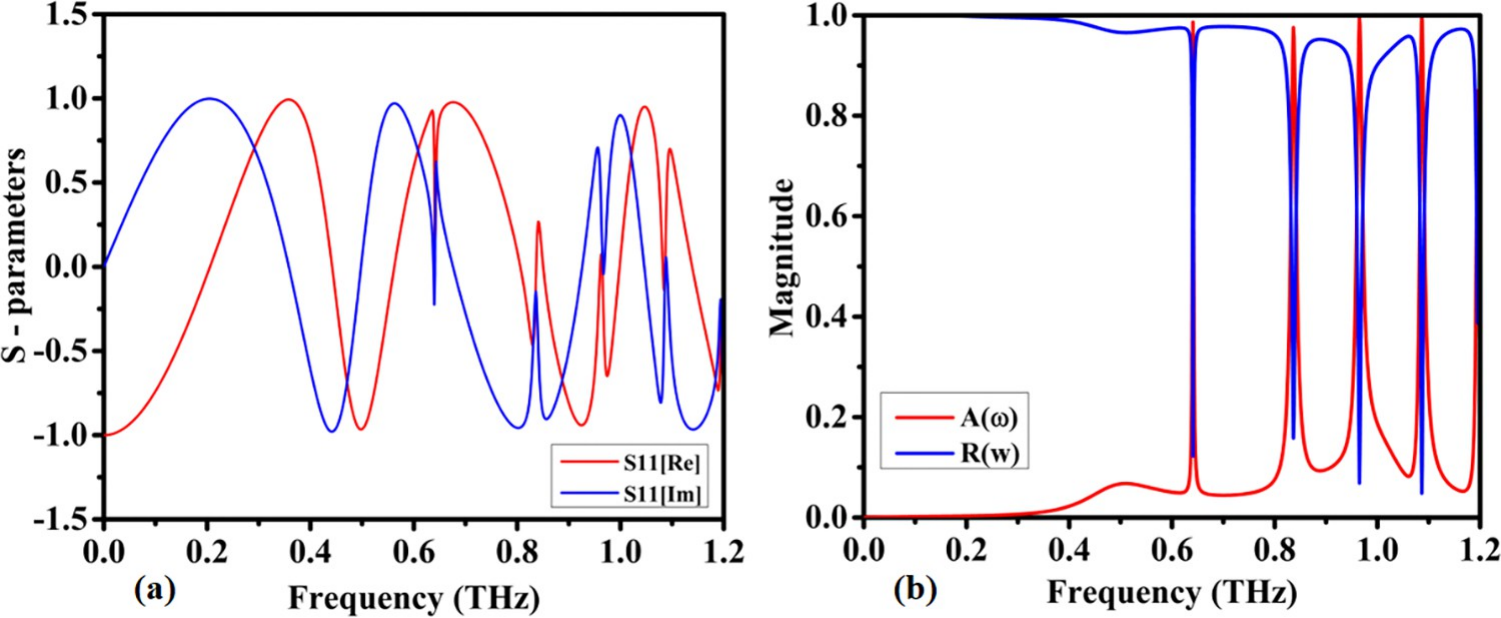
(a) S11 real and imaginary parts, (b) Reflection and absorption spectra of the absorber.

Metamaterial absorbers are characterized by their input reflection coefficient (S11), which consists of real and imaginary components that serve as vital parameters of performance. These components are illustrated in Fig 7A. The real part of S11 represents the resistive part of the material, while the imaginary part denotes the reactive part. At the resonant frequencies of the absorber, the real part of S11 approaches zero, indicating minimal reflection, whereas the imaginary part undergoes a sharp transition [93,94]. This behavior is key for determining the effectiveness of the absorber. For a metamaterial absorber to be highly efficient, it is essential to minimize the real part of S11. This minimization ensures that there is minimal reflection of incident electromagnetic waves, leading to better absorption. Additionally, the imaginary part of S11 should be matched to the impedance of free space, which is 377 Ω, at the desired frequencies [95,96]. This impedance matching is critical because it ensures that the electromagnetic energy is effectively dissipated within the absorber rather than being reflected back. The relationship between the real and imaginary parts of S11 and its magnitude (|S11|) can be expressed through the following equations [97].


Re(S11)=|S11|cos(θ) (1)

$$\mathrm{Img}(S_{11})=|S_{11}|\sin(\theta )$$ (2)

In these equations, θ represents the phase angle of S11. These relations enable the calculation of the real and imaginary components from the magnitude of S11, offering a comprehensive understanding of the absorber’s characteristics. By examining these components, valuable insights into the absorption properties of the metamaterial can be obtained. This understanding is vital for designing efficient absorbers that minimize reflections at the desired frequencies. By finely tuning these parameters, a metamaterial absorber can achieve optimized performance.

Fig 7B displays the reflection and absorption spectra of the metamaterial-based micro-biosensor. The quintuple-band nature is evident from the multiple distinct absorption peaks across the 0 to 1.2 THz range. These peaks result from the resonant coupling between the electromagnetic waves and the engineered structure of the metamaterial. In the proposed model, four distinct absorption peaks are recognized at nearly 0.6408 THz, 0.8365 THz, 0.965 THz, 1.0865 THz, and 1.195 THz, each exhibiting absorption capacities exceeding 98.5%, 97.5%, 99.5%, 99.7%, and 85%, respectively. These peaks signify highly efficient electromagnetic wave absorption at these frequencies.

The high absorption at specific frequencies is due to the perfect impedance matching between the metamaterial and free space. This minimizes reflection and maximizes energy transfer into the structure. The metamaterial’s design features, such as periodic patterns and subwavelength resonators, create localized surface plasmon resonances. These resonances enhance field confinement, leading to increased interaction with the target analytes. Each absorption peak corresponds to a resonant mode of the structure, which can be fine-tuned by adjusting the geometric parameters of the metamaterial. This tunability allows the sensor to be tailored for specific applications, such as detecting different biochemical markers. The sharpness and position of the peaks indicate the sensor’s sensitivity and selectivity, making it highly effective for multi-frequency analysis in biosensing applications.

### 4.2 Material properties and refractive index

**Fig 8:**
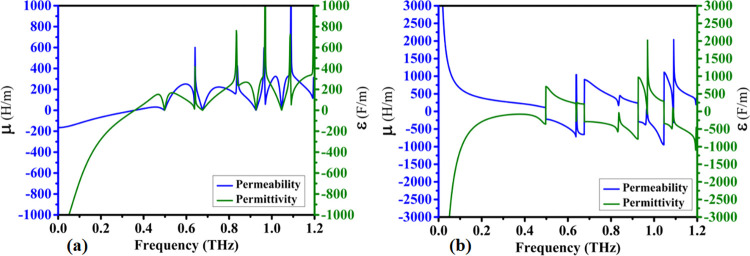
Real and imaginary components of Permeability (μ) and Permittivity (ε) for the proposed absorber: (a) Real components of μ and ε, (b) Imaginary components of μ and ε.

**Fig 9:**
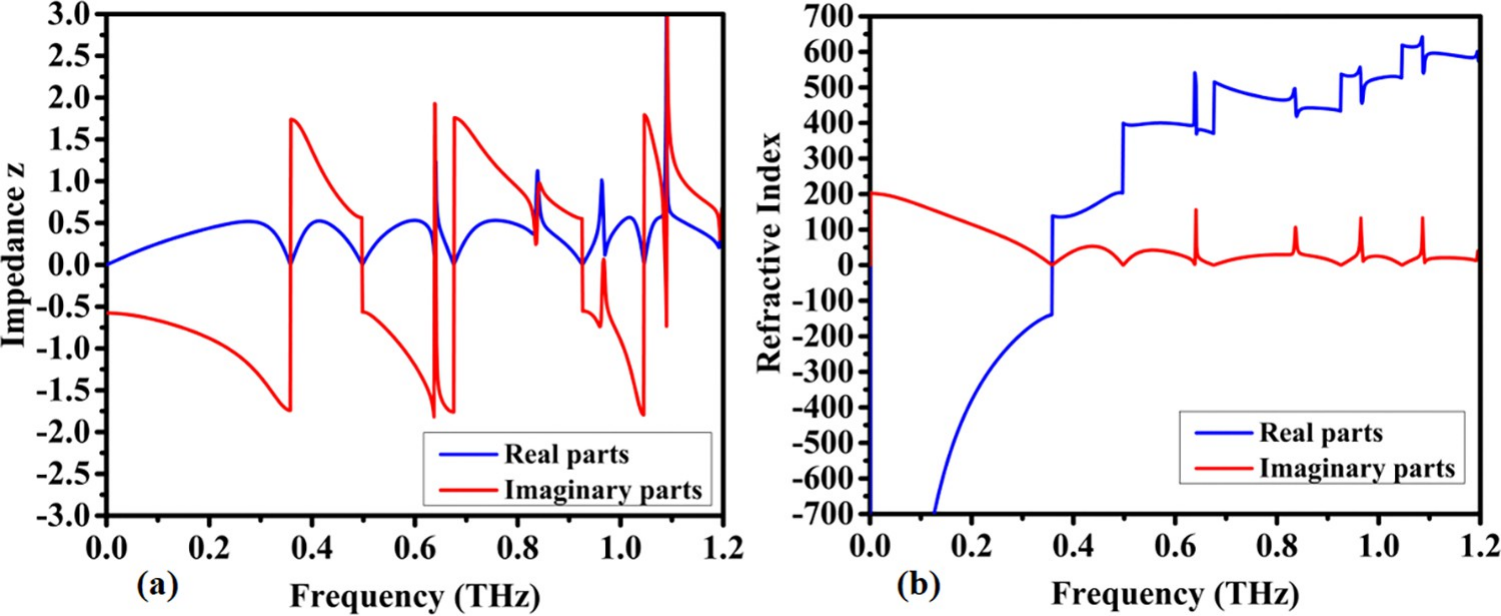
Simulated parameter responses of the proposed model: (a) Impedance (z), (b) Real and imaginary parts of the Refractive Index.

With the successful optimization of the structural design and after careful consideration of the material characteristics in its selection it is possible to focus on the final design and obtain the effective electromagnetic properties of the proposed metamaterial. The resulting real parts of the effective metamaterial permeability and permittivity are provided in Fig 8A while its imaginary parts are shown in Fig 8B. Directing the analysis to the higher frequency parts over 0.5 THz it is possible to observe that the metamaterial presents a very important behavior in its electromagnetic properties, it starts with very mild and continuous changes in its permeability and permittivity, but when it reaches the main resonances and absorbance peaks designed in the previous sections, the sensor reveals strong and steep changes. It is of strong interest to observe the responses shown in Fig 8B, especially for the imaginary magnetic part, where it also reveals linked absorbance characteristics to the resonance peaks, which can be attributed to a magnetic metamaterial absorber behavior. This property of some metamaterials constitute an important class of devices that have been highly desirable in several applications with the need of elevated frequency selectivity, as in sensors, which is the main target of the present work. Fig 9A presents the calculated impedance of the device, while Fig 9B shows the real and imaginary parts of the complex refractive index. The design focused on maximizing the absorption by reducing the reflection coefficient using impedance matching in the regions of interest near the quintuple absorption peaks. The imaginary part of Fig 9B also clearly shows elevated absorption values in these same regions.

### 4.3 Electric field (E-field) distributions

**Fig 10:**
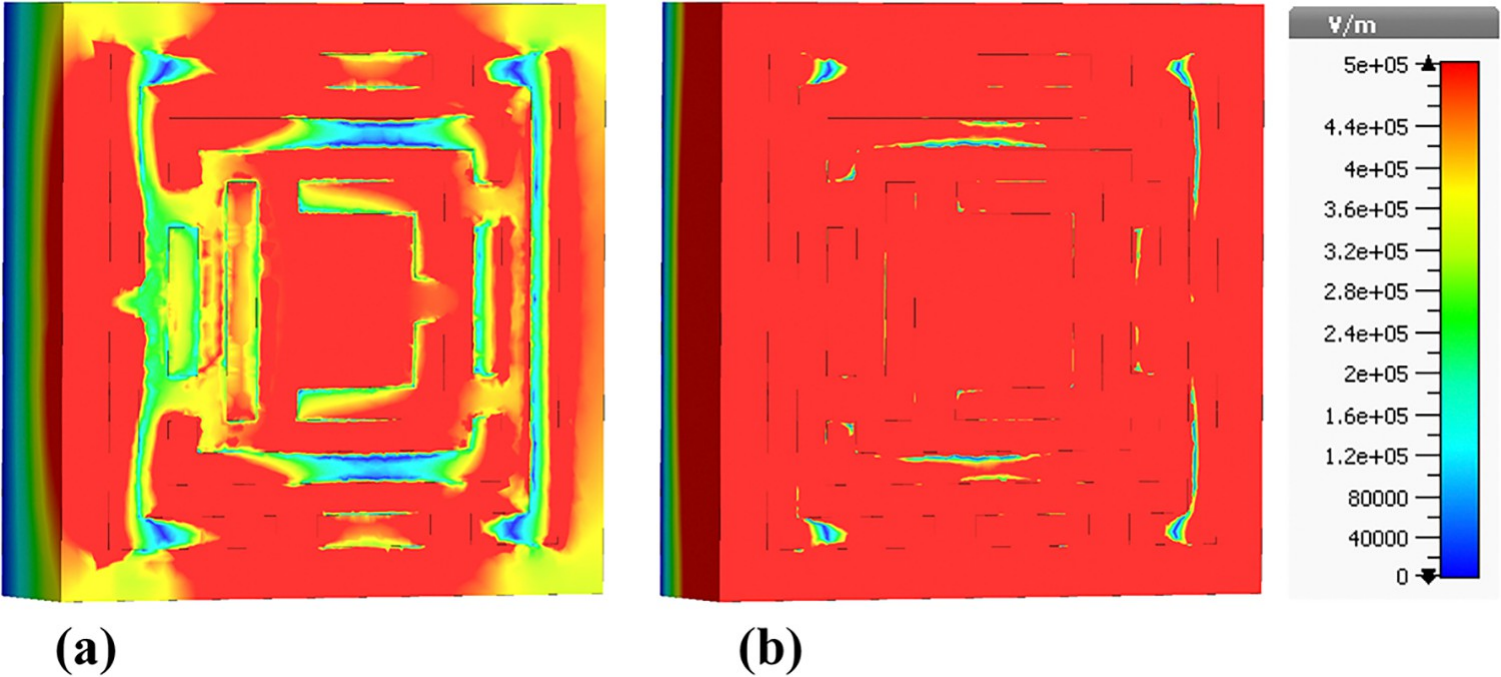
Visualization of the /E/-field distributions in the metamaterial structure at 0.6408 THz: (a) Real components, (b) Imaginary components.

**Fig 11:**
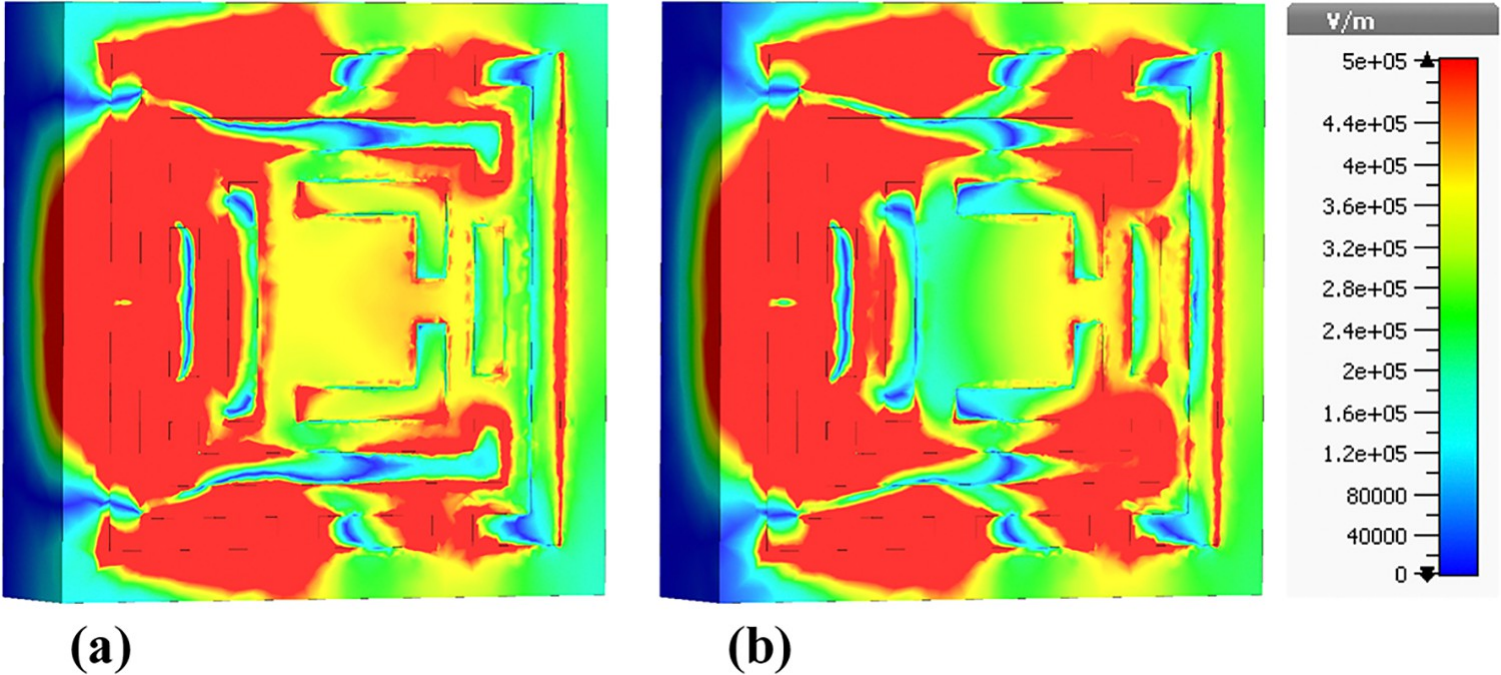
Visualization of the /E/-field distributions in the metamaterial structure at 0.8365 THz: (a) Real components, (b) Imaginary components.

**Fig 12:**
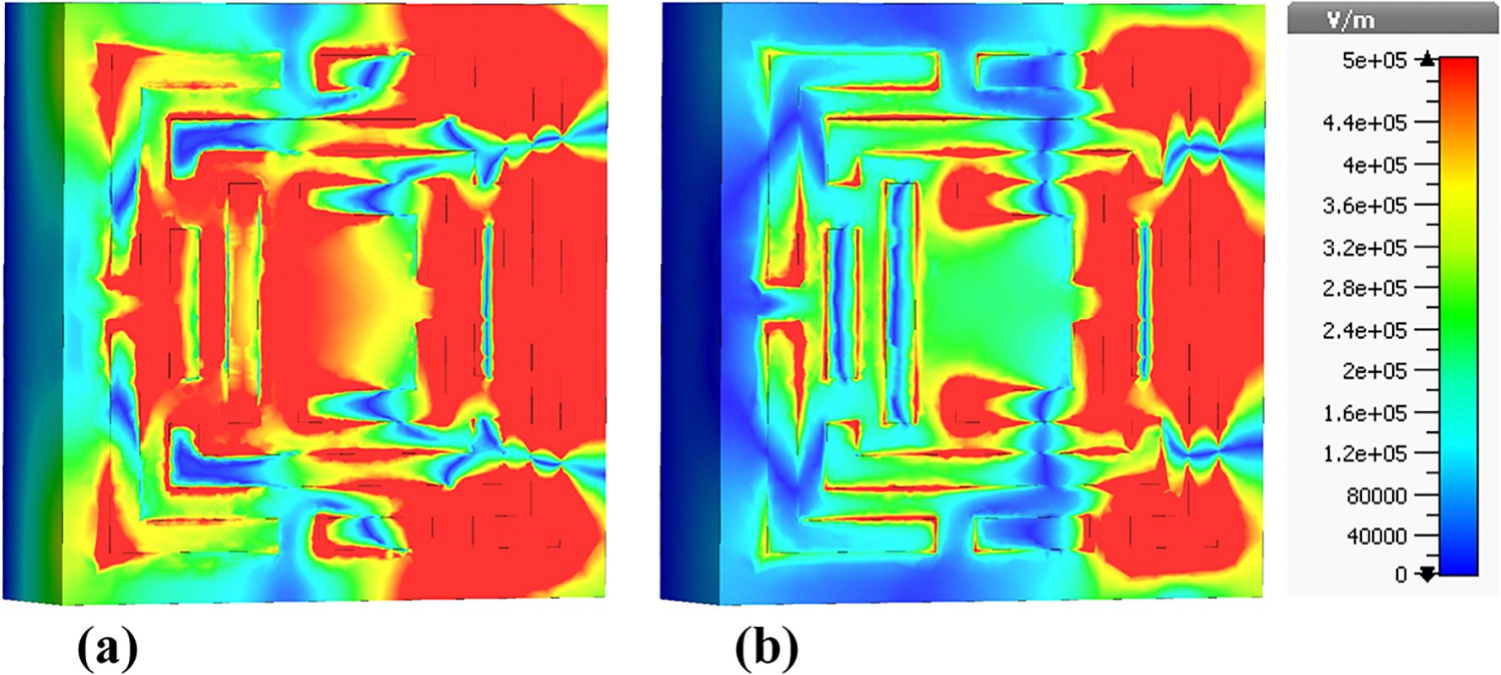
Visualization of the /E/-field distributions in the metamaterial structure at 0.965 THz: (a) Real components, (b) Imaginary components.

**Fig 13:**
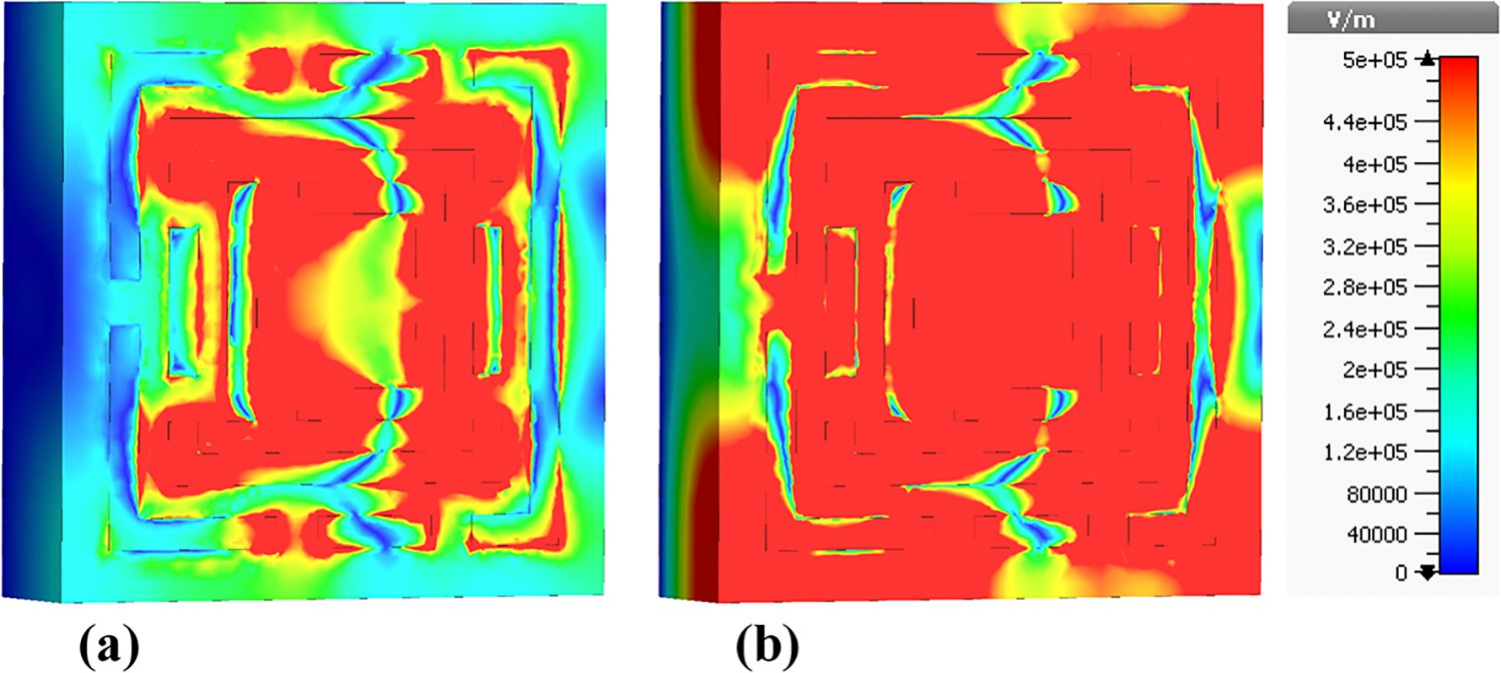
Visualization of the /E/-field distributions in the metamaterial structure at 1.0865 THz: (a) Real components, (b) Imaginary components.

**Fig 14:**
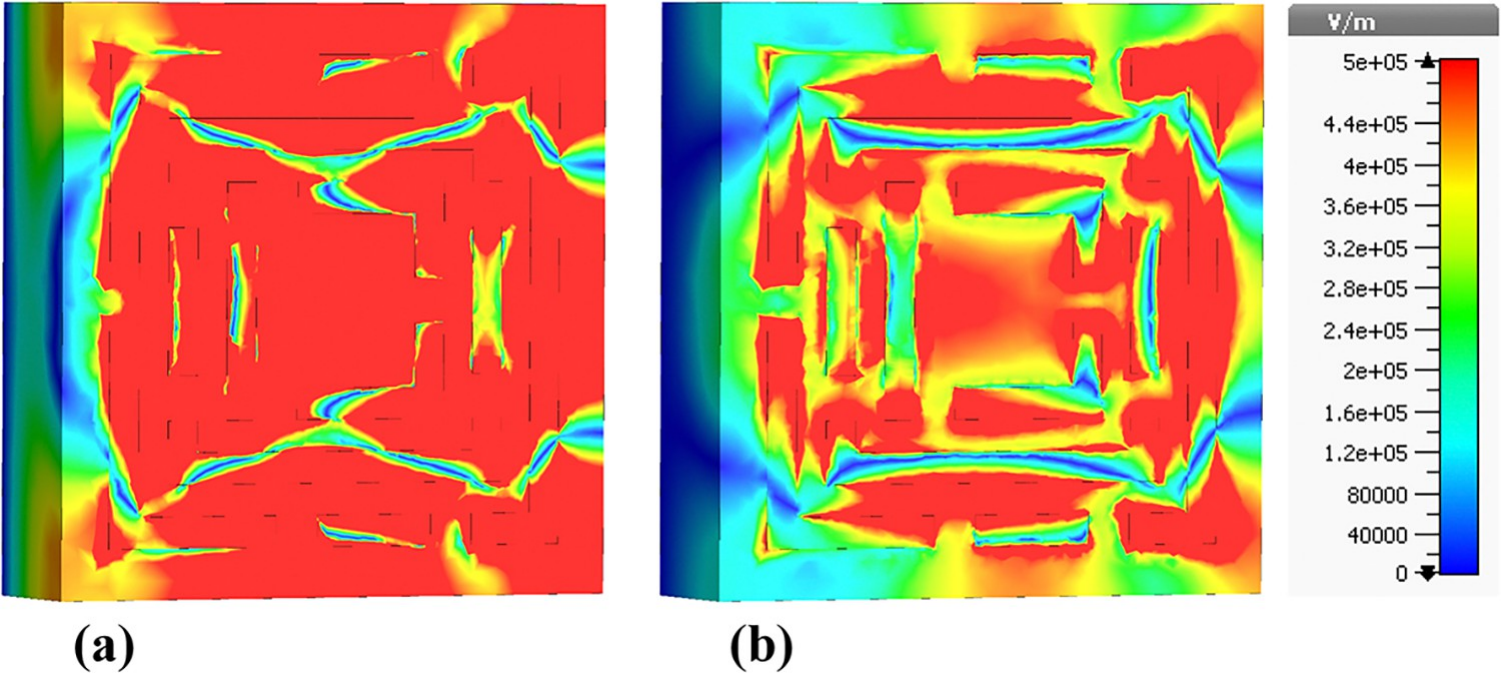
Visualization of the /E/-field distributions in the metamaterial structure at 1.195 THz: (a) Real components, (b) Imaginary components.

The following sections focus on the evaluation of the field distributions over the structure of the designed metamaterial based biosensor. Fig 10 shows a set of colormaps illustrating the intensity of the electric field at the frequency of 0.6408 THz, which corresponds to the first absorption peak. Fig 10A shows the real part while Fig 10B presents the imaginary part. The distribution on the real part shows that for this lower frequency band the outer structures are the most relevant in this resonator behavior. Focusing on the next absorbance peak, Fig 11 shows the real and imaginary electric field intensities for the frequency of 0.8365 THz. In this case the resonance is quite different from the previous case, being mainly from the lateral part of the D shaped ring, but also showing very high field intensities justifying the quality of the absorption obtained. Fig 12 presents the visualization of the electric field intensity for 0.965 THz, the third absorption peak. This is also linked to the lateral part of the D shaped ring and conclude the main contributions of the resonator provided by the structures inherited from Model 3. Fig 13 demonstrates the E-field distributions in the biosensor at 1.0865 THz. The real component (Fig 13A) shows high-intensity regions (red) along the metamaterial structure, indicating strong resonant interactions crucial for capturing and manipulating electromagnetic waves, enhancing the sensor’s sensitivity for detecting minute environmental changes. The imaginary component (Fig 13B) reveals phase-related characteristics with varying colors indicating different phase shifts within the structure. Finally, the results for the electric field intensity in the last absorption peak are provided in Fig 14 for the frequency of 1.195 THz. In this case there is a mixed scenario and most of the responses are due to the interaction of the several smaller structural components previously shown, but mainly linked to the inner rings. These diverse electric field colormaps provide relevant insights into the resonances working in the metamaterial and helped to understand the correlation between the structure and its performance. These analyses considerably contributed to the optimization process and were crucial to obtain the unique biosensor performance achieved in this work.

### 4.4 Magnetic field (H-field) distributions

The presented figures illustrate the H-field distributions within a metamaterial-based micro-biosensor designed for terahertz frequencies ranging from 0 THz to 1.2 THz, showcasing the real and imaginary components of the magnetic field intensity at specific frequencies: 0.6408 THz, 0.8365 THz, 0.965 THz, 1.0865 THz, and 1.195 THz. These visualizations highlight the quintuple-band nature of the biosensor, essential for its effectiveness in biomedical applications, particularly in early-stage cancer detection.

**Fig 15:**
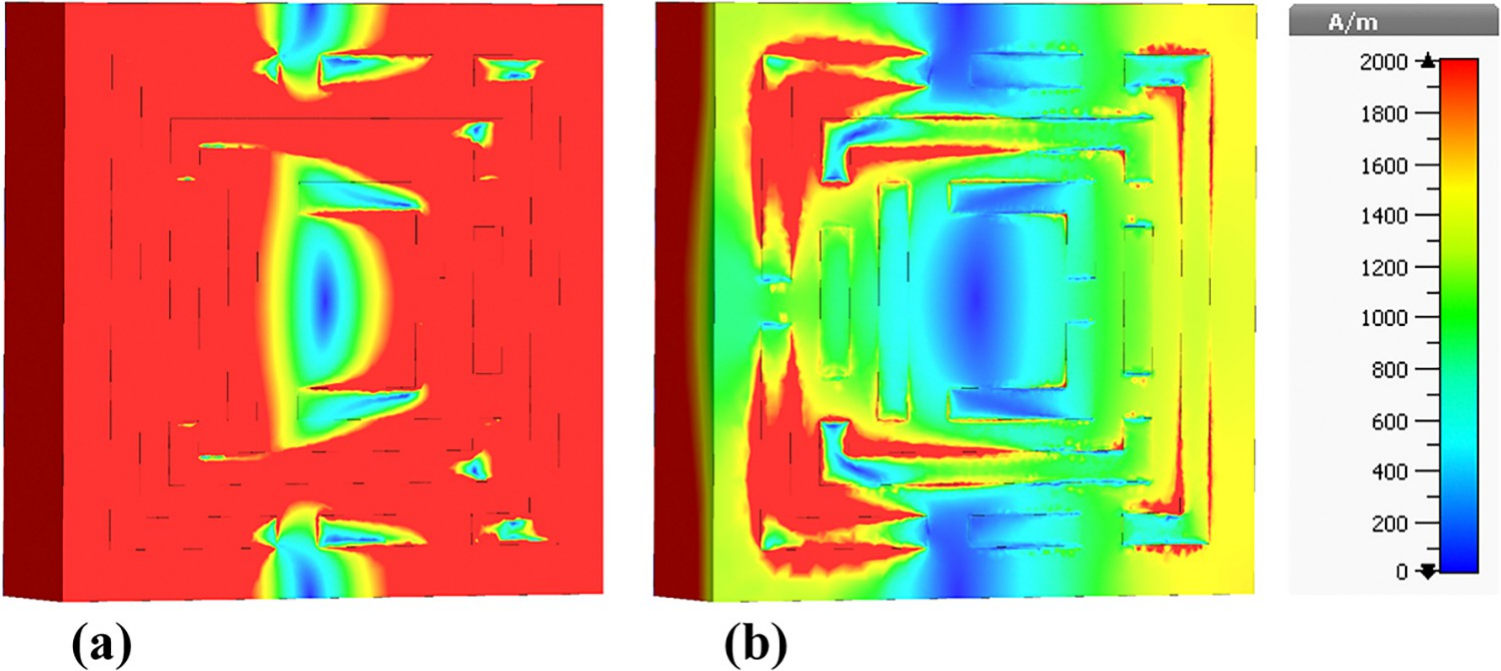
Visualization of the /H/-field distributions in the metamaterial structure at 0.6408 THz: (a) Real components, (b) Imaginary components.

Fig 15 depicts the H-field distribution at 0.6408 THz, illustrating the real Fig 15A and imaginary Fig 15B components. The color gradient from blue (0 A/m) to red (2000 A/m) indicates the intensity of the magnetic field. The high-intensity regions (red areas) correspond to localized resonances within the metamaterial structure, where the magnetic field is highly concentrated. These regions are critical for the sensor’s sensitivity, as they represent areas where the interaction with biomolecules is maximized.

**Fig 16:**
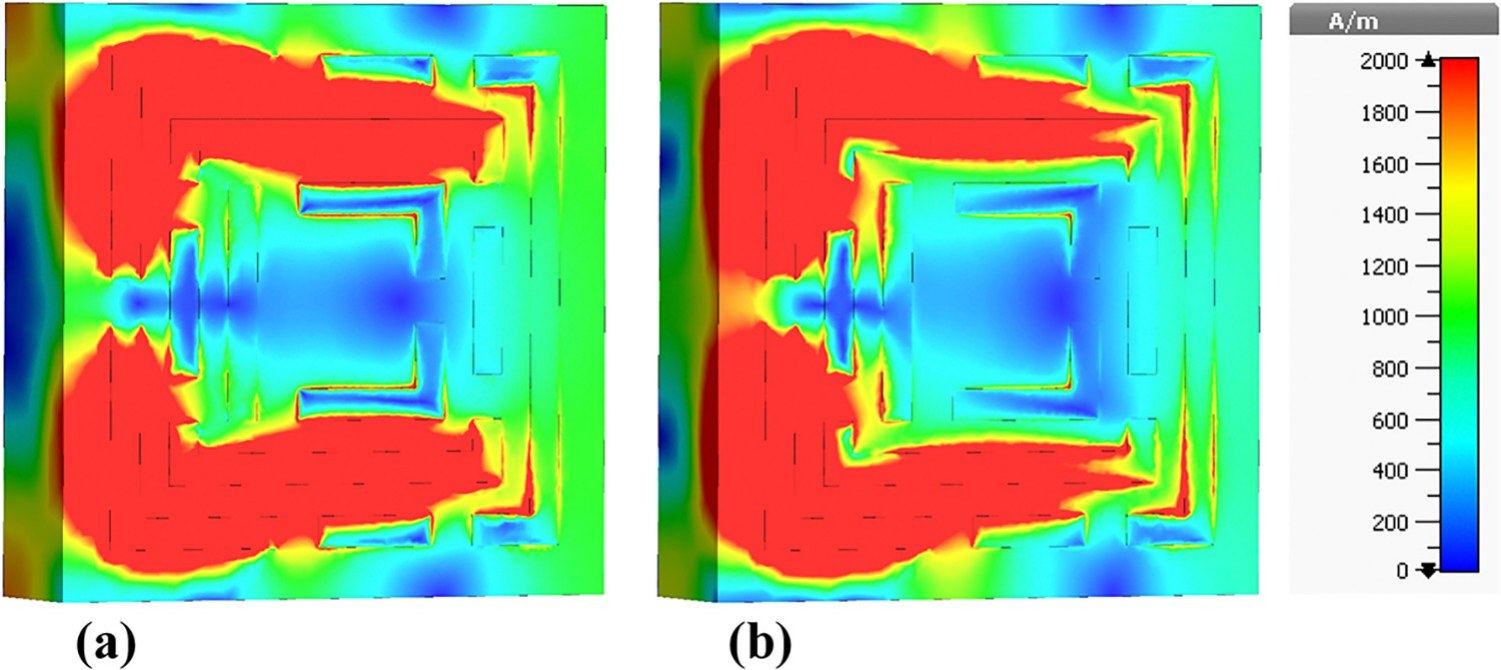
Visualization of the /H/-field distributions in the metamaterial structure at 0.8365 THz: (a) Real components, (b) Imaginary components.

Fig 16 presents the H-field distribution at 0.8365 THz. Similar to Fig 15, it shows the real Fig 16A and imaginary Fig 16B components with the same color gradient. The pattern of high-intensity regions is different, reflecting the change in resonance behavior at this frequency. The differences in the distribution patterns between Figs 15 and 16 suggest variations in the sensor’s response to different frequencies, highlighting the importance of the quintuple-band characteristic.

**Fig 17:**
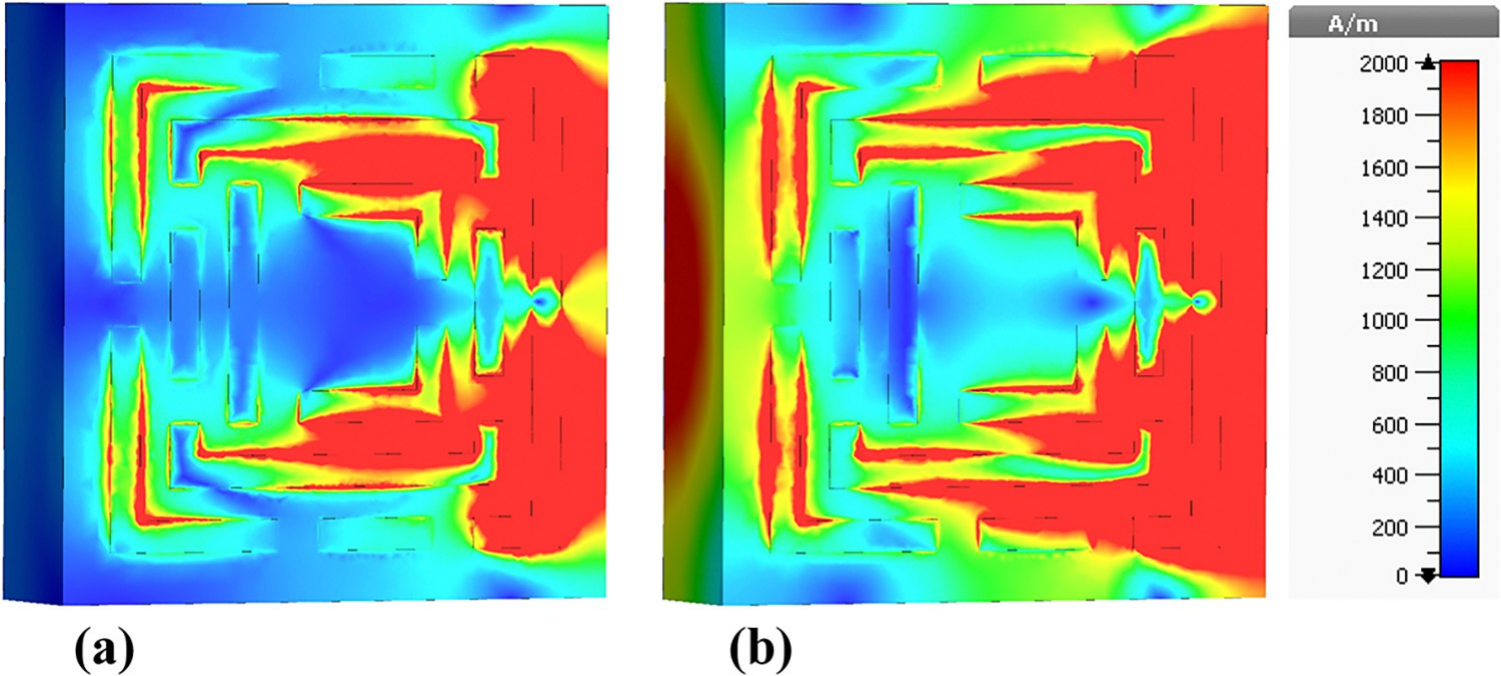
Visualization of the /H/-field distributions in the metamaterial structure at 0.965 THz: (a) Real components, (b) Imaginary components.

Fig 17 shows the H-field distribution at 0.965 THz, again with the real and imaginary components. The intensity distribution in this figure exhibits unique patterns compared to the previous figures, indicating another resonant frequency of the biosensor. The consistent appearance of high-intensity regions across different frequencies confirms the sensor’s capability to operate effectively across a wide frequency range.

The color variations in these figures arise from the interaction of the electromagnetic waves with the metamaterial structure. The high-intensity (red) areas indicate strong localization of the magnetic field due to resonant effects, which are essential for enhancing the sensor’s sensitivity. The blue areas represent low magnetic field intensity, indicating regions of minimal interaction.

The differences between the figures reflect the sensor’s multi-band resonance capability. Each figure shows distinct patterns of high and low-intensity regions, corresponding to different resonant frequencies. These variations are crucial for the biosensor’s design, as they enable the detection of a broad range of biomolecular signatures at different frequencies, enhancing the sensor’s versatility and effectiveness.

**Fig 18:**
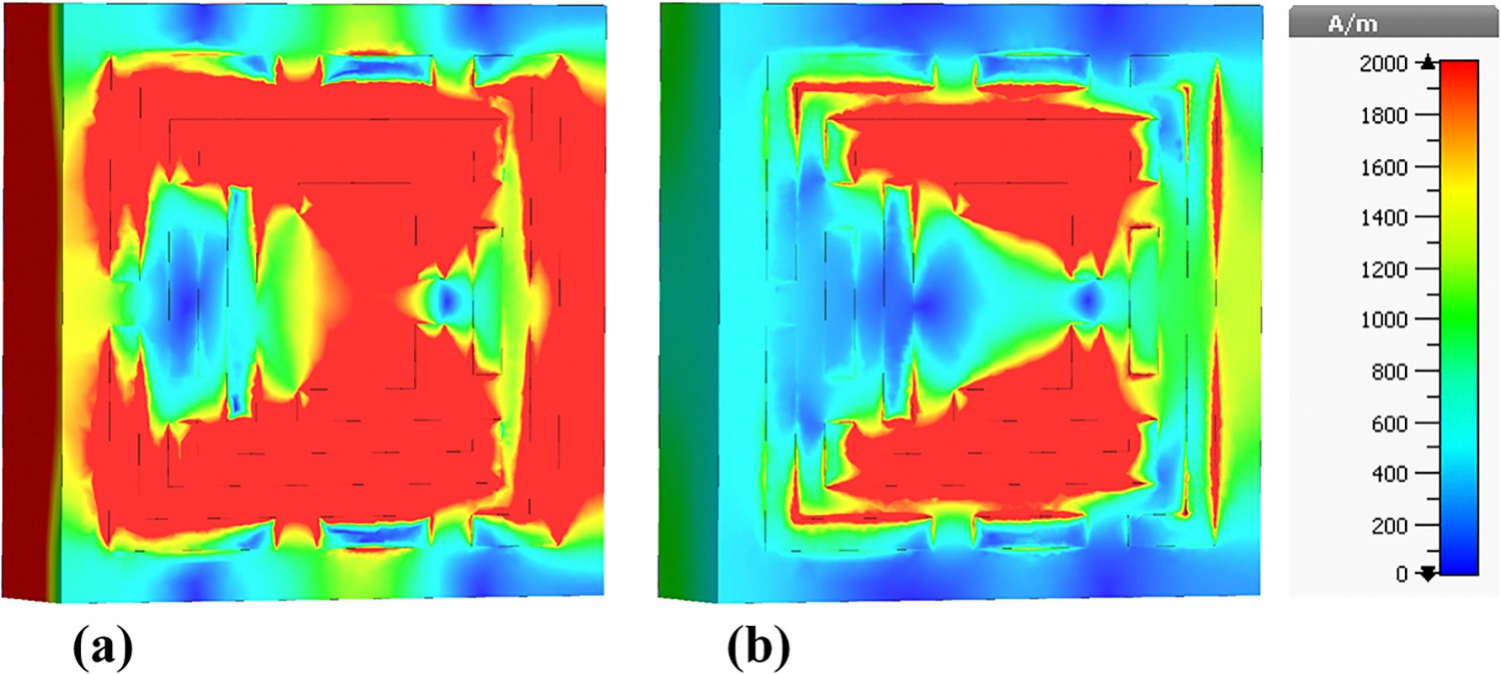
Visualization of the /H/-field distributions in the metamaterial structure at 1.0865 THz: (a) Real components, (b) Imaginary components.

Fig 18 illustrates the H-field distribution within the biosensor at a frequency of 1.0865 THz. In Fig 18A, the real component shows high-intensity regions predominantly around the edges and specific internal segments of the metamaterial. These red areas signify strong magnetic field localization, which is essential for enhancing the sensor’s sensitivity to target biomolecules. The high-intensity regions correspond to the resonant modes of the metamaterial structure, where the electromagnetic waves are strongly confined, resulting in increased interaction with the analyte.

Fig 18B displays the imaginary component of the H-field. The patterns in the imaginary component indicate variations in the phase and energy dissipation within the metamaterial. The differences in color patterns between the real and imaginary components highlight the complex nature of the electromagnetic interactions, where the imaginary component provides insights into the lossy behavior and energy absorption characteristics of the sensor.

**Fig 19:**
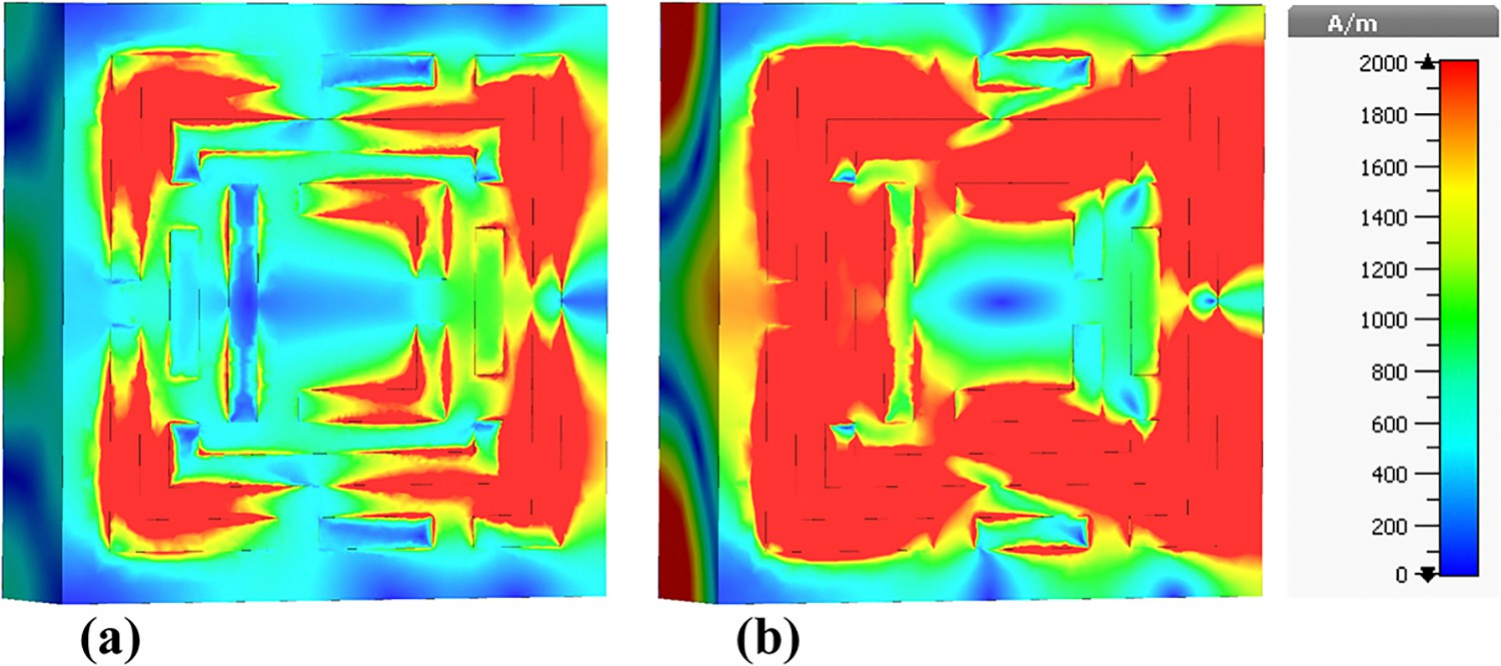
Visualization of the /H/-field distributions in the metamaterial structure at 1.195 THz: (a) Real components, (b) Imaginary components.

Fig 19 shows the H-field distribution at a slightly higher frequency of 1.195 THz. In Fig 19A, the real component reveals high-intensity regions concentrated in different parts of the metamaterial structure compared to Fig 18. These red areas, indicative of strong magnetic field localization, are crucial for the sensor’s sensitivity. The shift in high-intensity regions between Figs 18 and 19 suggests that the resonant modes and field confinement patterns vary with frequency, which is a characteristic feature of metamaterial-based sensors.

Fig 19B illustrates the imaginary component of the H-field at 1.195 THz. The color patterns here reflect the phase shifts and energy dissipation within the metamaterial. The distribution of colors, especially the red and yellow areas, indicates regions where energy absorption is significant, contributing to the sensor’s overall electromagnetic response.

The color patterns in both figures are a direct result of the magnetic field intensity distribution within the metamaterial at different frequencies. The red regions represent areas of high magnetic field intensity, while the blue regions indicate low intensity. These patterns arise due to the resonant behavior of the metamaterial, which causes electromagnetic waves to be strongly localized in certain regions at specific frequencies. The differences in color patterns between the real and imaginary components are due to the nature of the electromagnetic interactions, with the real component representing the field’s amplitude and the imaginary component reflecting phase and energy dissipation.

The primary difference between Figs 18 and 19 lies in the frequency at which the H-field distributions are visualized: 1.0865 THz and 1.195 THz, respectively. This change in frequency results in different resonant modes within the metamaterial structure, leading to variations in the localization and intensity of the magnetic fields. Consequently, the high-intensity regions (red areas) shift positions between the figures, indicating the sensor’s multi-band capability and its ability to respond to different frequencies, which is essential for detecting a wide range of biomolecular signatures.

Understanding the H-field distributions at various frequencies is crucial for optimizing the design of the biosensor. High-intensity regions signify strong electromagnetic interactions, which are vital for enhancing the sensor’s sensitivity to detect low concentrations of target biomolecules. This high sensitivity is particularly important for early cancer detection, as it enables the identification of biomarkers at the earliest stages of the disease, leading to timely diagnosis and treatment.

The high sensitivity of the biosensor is attributed to the strong localization of the magnetic field in the high-intensity regions, enhancing the interaction with target biomolecules. The quintuple-band nature of the biosensor, demonstrated by the distinct H-field patterns at multiple frequencies, allows for the detection of various biomolecular signatures associated with different types of cancer, including leukemia. The detailed visualization of the H-field distributions enables researchers to identify the most effective resonant frequencies and the corresponding sensor regions that maximize interaction with target biomolecules. This information is critical for designing biosensors with high sensitivity and specificity, which are essential for detecting leukemia at early stages. By understanding the electromagnetic behavior of the sensor across multiple frequencies, researchers can improve the sensor’s performance, making it a valuable tool in biomedical diagnostics and early cancer detection.

### 4.5 Surface current distributions and angular dependence of absorption

**Fig 20:**
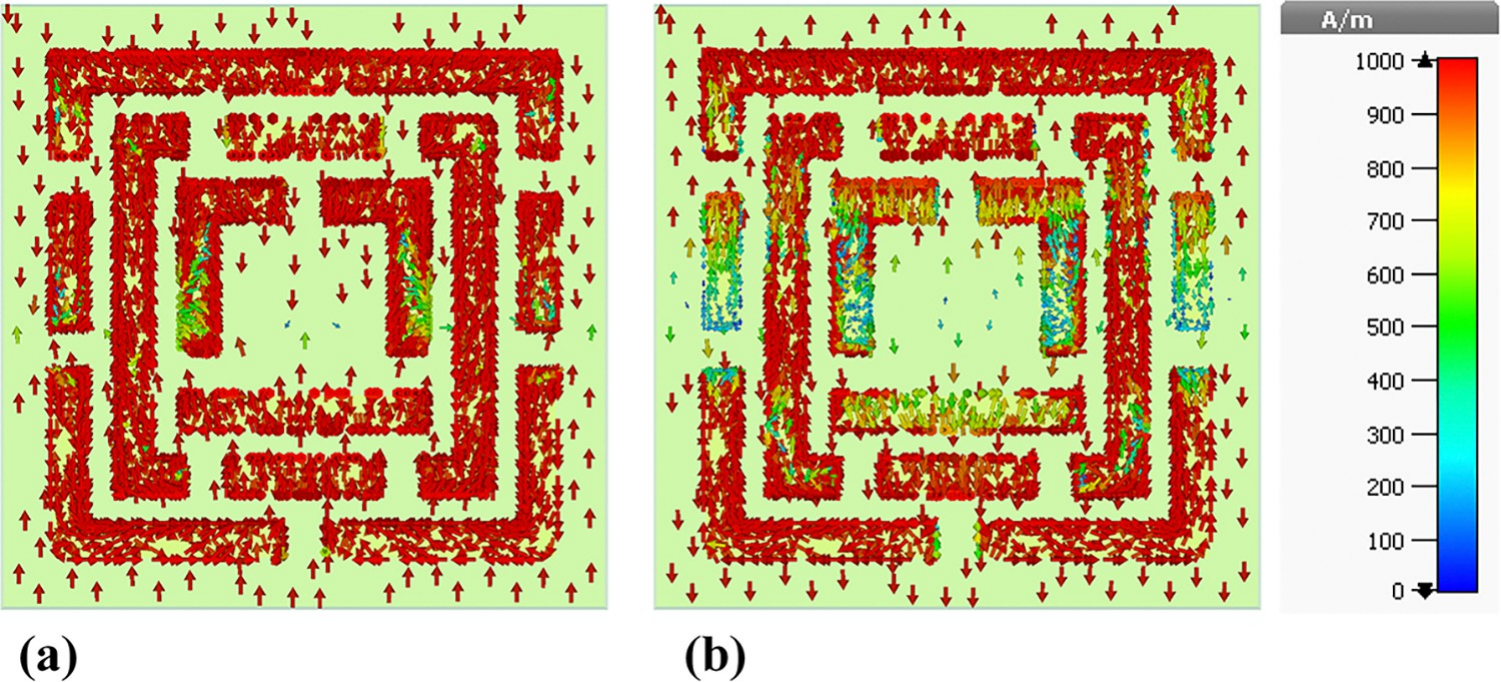
Visualization of the surface current distributions in the metamaterial structure at 0.6408 THz: (a) Real components, (b) Imaginary components.

**Fig 21:**
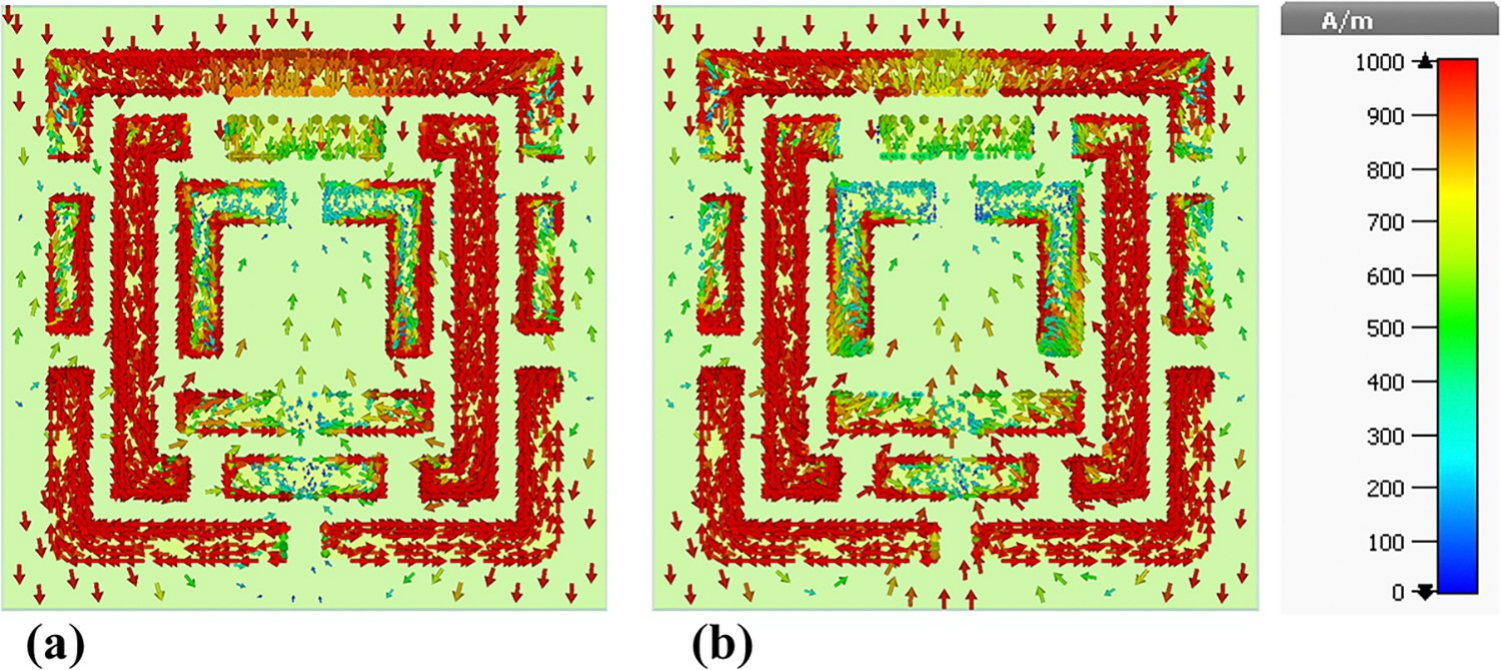
Visualization of the surface current distributions in the metamaterial structure at 0.8365 THz: (a) Real components, (b) Imaginary components.

**Fig 22:**
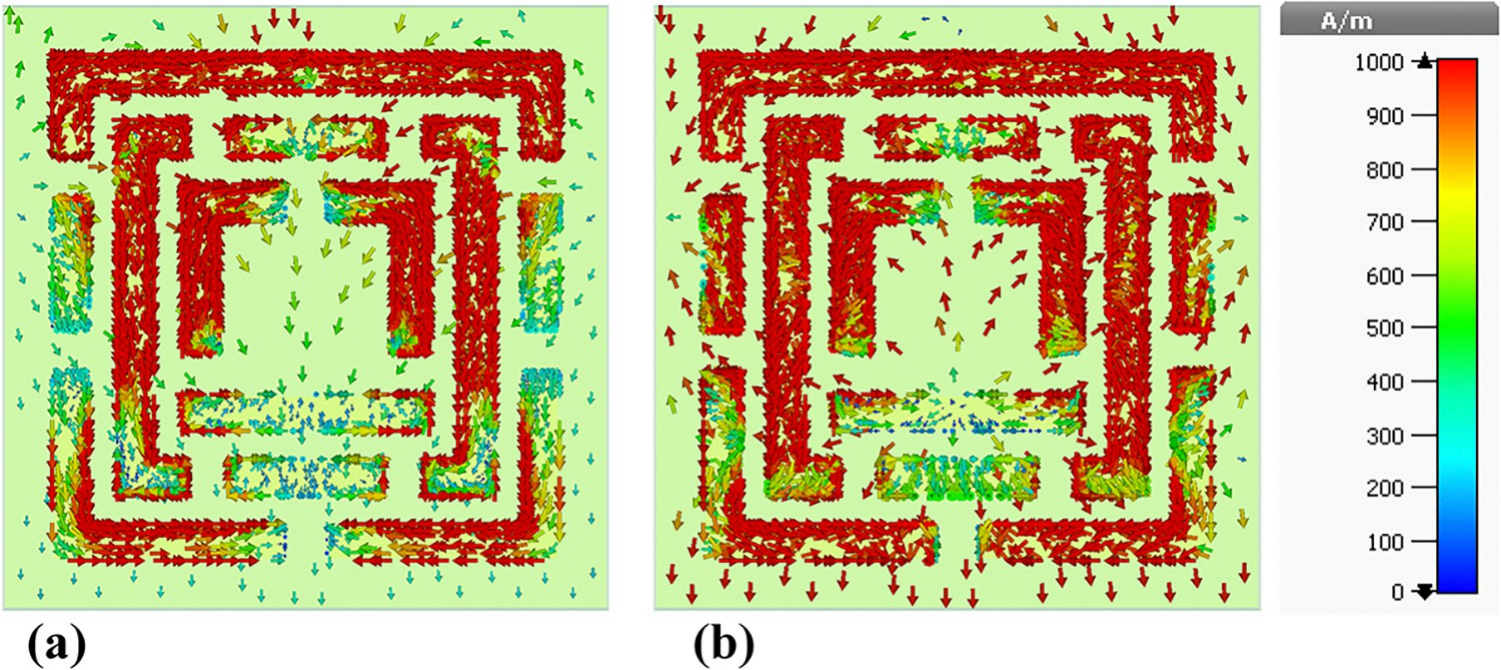
Visualization of the surface current distributions in the metamaterial structure at 0.965 THz: (a) Real components, (b) Imaginary components.

**Fig 23:**
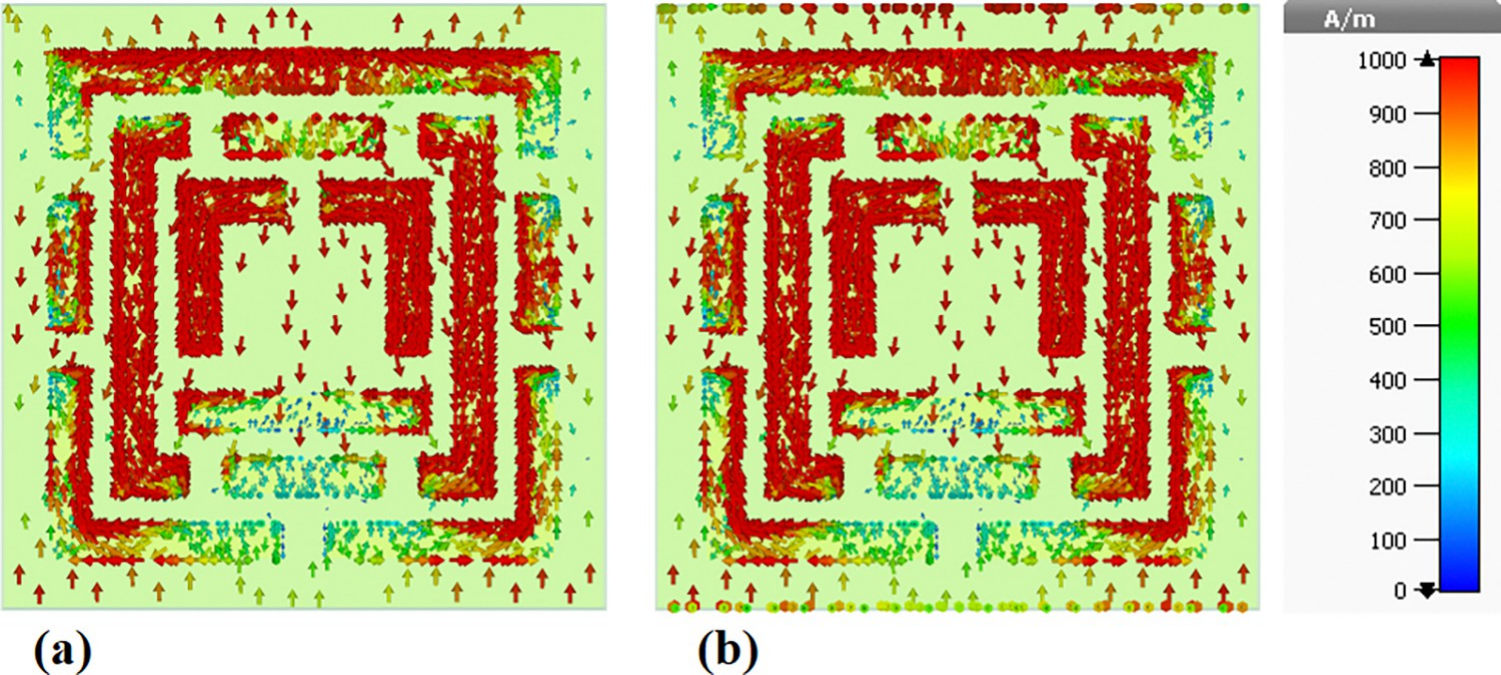
Visualization of the surface current distributions in the metamaterial structure at 1.0865 THz: (a) Real components, (b) Imaginary components.

**Fig 24:**
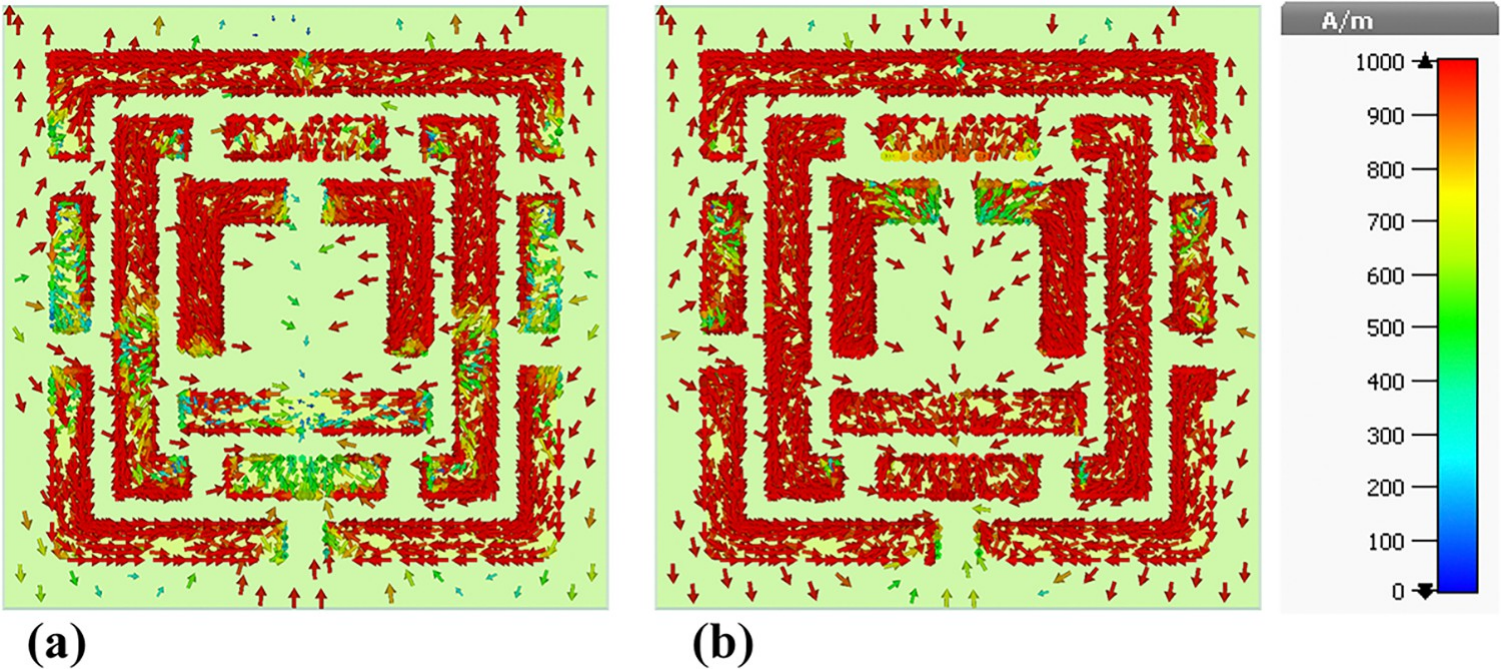
Visualization of the surface current distributions in the metamaterial structure at 1.195 THz: (a) Real components, (b) Imaginary components.

This sub-section presents a detailed analysis of the surface current density within the metamaterial-based micro-biosensor operating in the terahertz frequency range (0–1.2 THz). The quintuple-band biosensor’s response was investigated at five specific frequencies: 0.6408 THz, 0.8365 THz, 0.965 THz, 1.0865 THz, and 1.195 THz. The surface current density distribution, visualized in terms of real (Figs 20A, 21A, 22A, 23A and 24A) and imaginary (Figs 20B, 21B, 22B, 23B and 24B) components, provides insights into the sensor’s electromagnetic behavior.

The real component highlights areas of maximum magnetic field intensity (red regions) corresponding to strong localized currents. These regions, primarily concentrated along the intricate metamaterial structure, indicate efficient electromagnetic wave interaction. The imaginary component, characterized by color variations, reveals phase-related information within the metamaterial.

A comparative analysis across frequencies demonstrates the sensor’s frequency-dependent behavior. The intensity and distribution of currents vary significantly, reflecting the sensor’s ability to resonate at multiple frequency bands. For instance, a more uniform current distribution is observed at 0.6408 THz (Fig 20A), while a highly localized pattern is evident at 1.195 THz (Fig 24A).

Understanding the surface current density is crucial for optimizing the biosensor’s design. The interplay between the real and imaginary components provides valuable information about the electromagnetic interactions within the metamaterial structure. By correlating the surface current density with the sensor’s overall performance, researchers can identify optimal operating frequencies and make necessary design modifications to enhance sensitivity and specificity for target biomolecule detection.

The findings presented in this sub-section contribute to the development of advanced biosensing technologies with improved diagnostic capabilities. By understanding the frequency-dependent behavior of the surface current density and its relationship to the sensor’s performance, researchers can optimize the design for specific applications such as early cancer detection.

## 5. Blood cancer diagnosis

The proposed terahertz metamaterial biosensor offers several significant advancements in blood cancer diagnostics compared to existing techniques. Traditional methods, such as bone marrow biopsies and standard blood tests, are often invasive and can be limited in detecting cancer in its early stages. While recent innovations like liquid biopsies, which analyze circulating tumor DNA (CTDNA) and circulating tumor cells (CTCs), provide a less invasive option, they are still dependent on specific biomarkers that may not fully capture the complexity of disease progression in all cases [98,99]. Similarly, although Next-Generation Sequencing (NGS) has revolutionized cancer diagnostics by offering comprehensive genomic profiling and detecting minimal residual disease (MRD), it requires specialized laboratory environments and can be resource-intensive [100].

Our terahertz biosensor addresses these challenges by utilizing a quintuple-band design, which enables the detection of multiple biomarkers across five distinct frequency ranges between 0.6 to 1.2 THz. This capability significantly enhances the accuracy of detection, making it more effective in identifying various cancer subtypes and distinguishing between healthy and cancerous cells. Unlike liquid biopsies or NGS, which focus on molecular or genetic mutations, our biosensor relies on terahertz absorption characteristics to detect subtle changes in the refractive index and electric field distribution, providing a highly sensitive and non-invasive diagnostic tool. This ability to detect abnormalities early, even at minimal disease levels, is particularly crucial for blood cancer diagnosis, where early intervention can significantly improve treatment outcomes.

Additionally, the biosensor achieves an absorption rate of over 95% across all five bands, ensuring high sensitivity and precision in cancer detection. Its high Figure of Merit (FOM) and Quality Factor (Q-Factor) values further underline its efficiency, allowing it to detect even the smallest variations in biological tissue. The quintuple-band design also offers the advantage of monitoring multiple biomarkers concurrently, which can improve the overall diagnostic accuracy and provide greater flexibility for detecting various forms of blood cancer. Furthermore, the sensor’s ultra-compact structure makes it highly suitable for integration into real-time monitoring systems and portable diagnostic devices, thus enhancing its practical utility in both clinical and point-of-care settings. The proposed biosensor not only complements but also surpasses many current diagnostic methods by providing a highly sensitive, non-invasive, and multi-band detection platform for early-stage blood cancer. Its innovative design and superior performance metrics position it as a promising solution for enhancing the accuracy and efficiency of blood cancer diagnostics.

**Fig 25:**
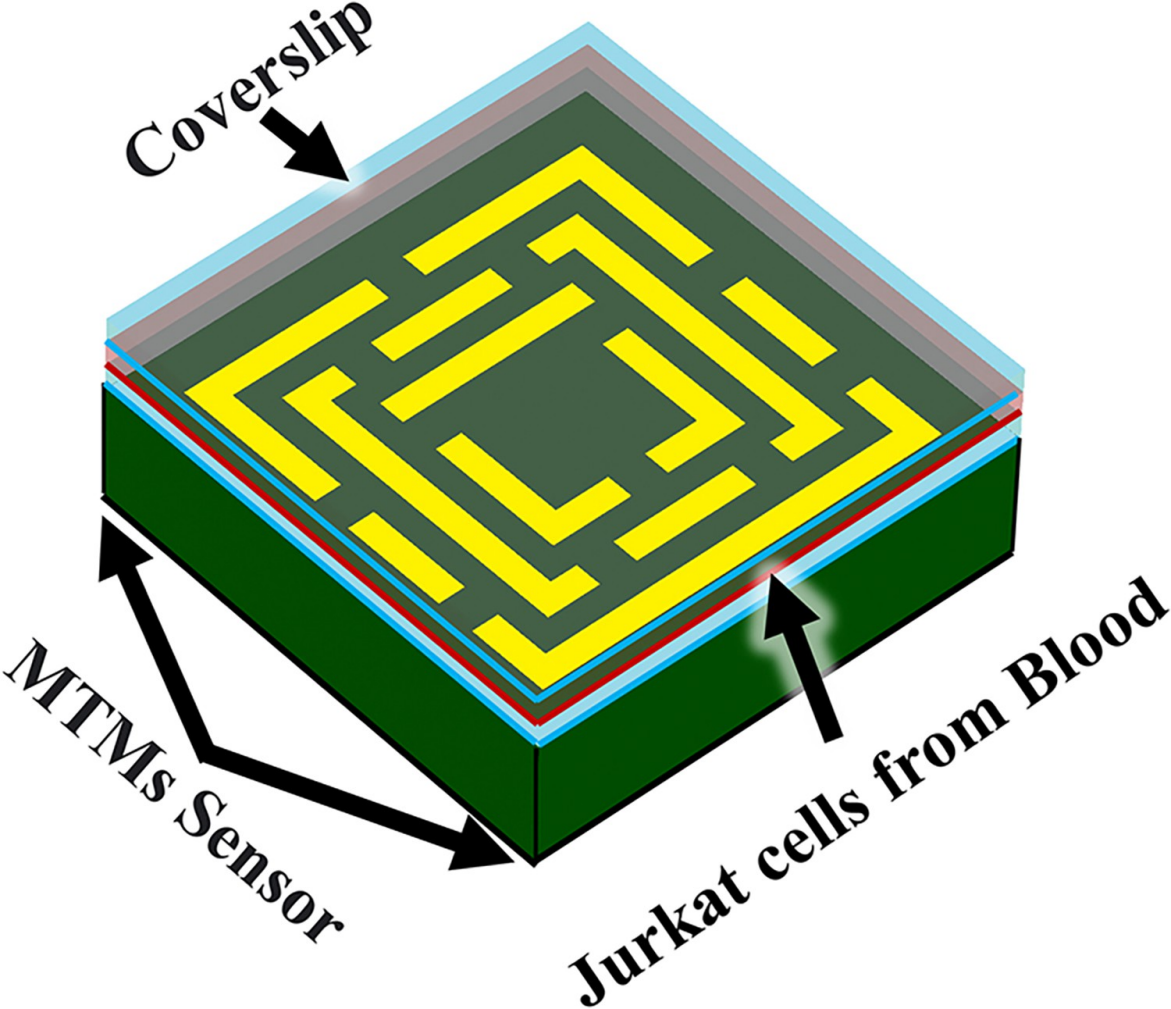
Analysis of the absorption coefficient of the proposed biosensor in both healthy blood and blood affected by cancer.

Fig 25 displays a 3-D schematic of the proposed terahertz biosensor, which is composed of an MTM waveguide and a coverslip with Jurkat cells from blood. The coverslip plays a crucial role in the performance of the proposed terahertz metamaterial-based biosensor. Its geometrical parameters, such as thickness and refractive index, significantly influence the sensor’s absorption properties and detection capabilities.

By optimizing these parameters, it is possible to enhance light-matter interaction, leading to higher absorption and improved sensitivity. The coverslip can also act as a resonant cavity, improving the biosensor’s sensitivity to specific biomarkers. Additionally, it provides mechanical support and helps to minimize reflections and scattering of terahertz waves.

Using a coverslip can prevent contamination and simplify the cleaning process, ensuring accurate measurements and reducing the need for extensive sensor cleaning. By carefully considering the coverslip’s geometrical parameters and its use in conjunction with the biological sample, the overall performance of the biosensor can be optimized.

The structural parameters are provided in Table 1. In the center of structure two types of bloods are injected. In healthy blood, biosensors can be utilized to measure levels of various indicators such as glucose, cholesterol, and electrolytes, providing valuable information about an individual’s overall health status.

When it comes to blood affected by cancer, biosensors can play a crucial role in detecting cancer-specific biomarkers like circulating tumor cells, tumor-related proteins, or genetic mutations. These biomarkers can help in early cancer diagnosis, monitoring disease progression, and assessing treatment effectiveness of the hexagonal cavity and the MIM waveguide.

Normal blood and blood cancer have different frequencies in the electromagnetic spectrum. Normal blood typically has a frequency range of around 0.8 to 0.9 THz, while blood cancer cells can have frequencies that range from 1.1 to 1.2 THz. This difference in frequencies can be used in medical imaging techniques like terahertz spectroscopy to differentiate between healthy and cancerous blood cells.

The assertion that normal blood and blood cancer cells exhibit distinct frequency ranges within the terahertz (THz) spectrum is based on preliminary research and theoretical models. These differences in frequency are attributed to the altered dielectric properties and dispersive behavior of blood cancer cells, which result from changes in their molecular composition and water content.

Studies have shown that cancerous cells have a higher refractive index than normal cells, with values ranging from 1.376 for normal blood to 1.390 for blood cancer cells [71,101–103]. This difference in refractive index is likely due to changes in the molecular composition and structure of cancer cells, which can alter their interaction with electromagnetic waves.

The altered dielectric properties of cancer cells can result in shifts in their characteristic absorption frequencies within the THz range. These frequency shifts can be detected and analyzed using THz spectroscopy, allowing for the differentiation between healthy and cancerous blood cells.

While the specific frequency ranges mentioned in the previous few paragraphs (0.8–0.9 THz for normal blood and 1.1–1.2 THz for blood cancer cells) are based on preliminary studies, the principle of using THz spectroscopy to differentiate between healthy and cancerous cells remains promising. Further research is needed to establish a definitive relationship between blood cell type and specific THz frequency signatures, taking into account the dispersive behavior of blood and the differences in refractive index between normal and cancerous cells, the ability to differentiate between normal and cancerous blood cells using THz spectroscopy is based on the differences in their dielectric properties and refractive indices. These differences arise from alterations in the molecular composition and structure of cancer cells, resulting in shifts in their characteristic absorption frequencies within the THz range.

**Fig 26:**
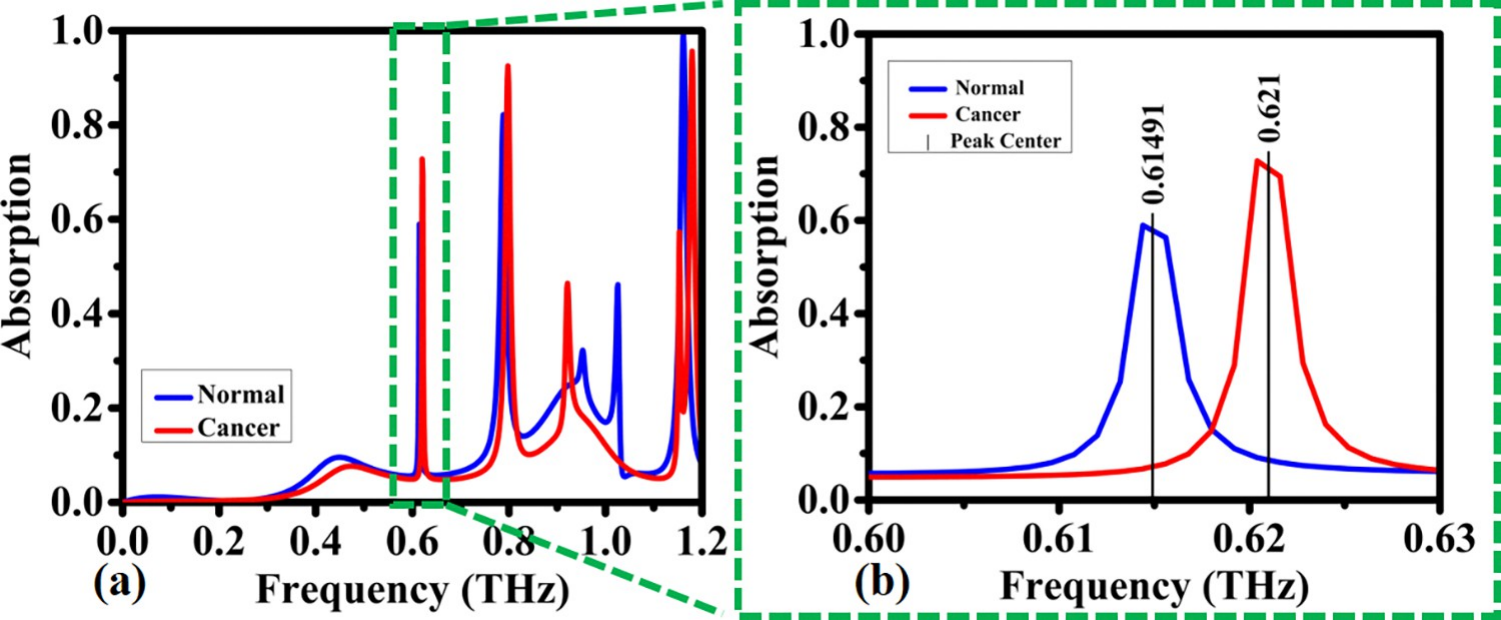
Detection of absorption coefficients by the proposed biosensor for normal blood and blood cancer within the frequency range of: (a) 0–1.2 THz, (b) 0.6–0.63 THz.

In order to benchmark of the structure and its effects on the sensing properties of the structure, we altered the range of input source frequency from 0.0 to 1.2 in steps of 0.2 for two bloods a) normal b) cancer. Fig 26A and 26B shows the relationship between the resonance frequency and the absorption for different blood for mode1 and mode2. As can be seen both blood in different frequencies have nearly perfect absorption. This mechanism is used for this biosensor.

**Fig 27:**
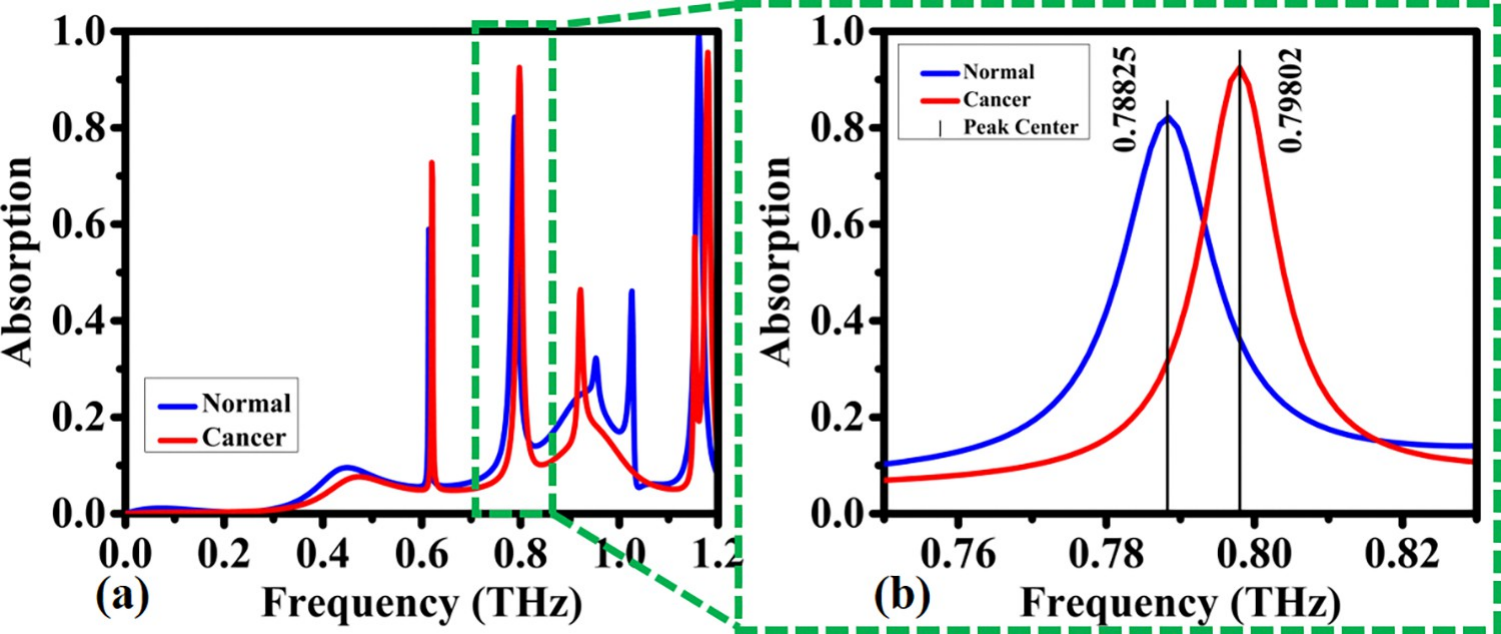
Detection of absorption coefficients by the proposed biosensor for normal blood and blood cancer within the frequency range of: (a) 0–1.2 THz, (b) 0.76–0.82 THz.

This structure as can be seen in Fig 27 is a quintuple-band biosensor. The quintuple-band terahertz cancer biosensor is a cutting-edge technology that utilizes terahertz radiation to detect and diagnose cancer at an early stage. This biosensor operates in five different frequency bands within the terahertz range, allowing for high sensitivity and accuracy in cancer detection. By analyzing the unique terahertz absorption signatures of cancerous tissue, this biosensor can provide quick and reliable results, leading to improved outcomes for patients. Such advanced biosensors hold great promise in the field of medical diagnostics and could revolutionize the way cancer is detected and treated. In second band the evaluation of the biosensor is also done. As can be seen in Fig 27B, in case of blood cancer the absorption is experienced blue shift from normal case and it is occurred in 0.8 THz.

**Fig 28:**
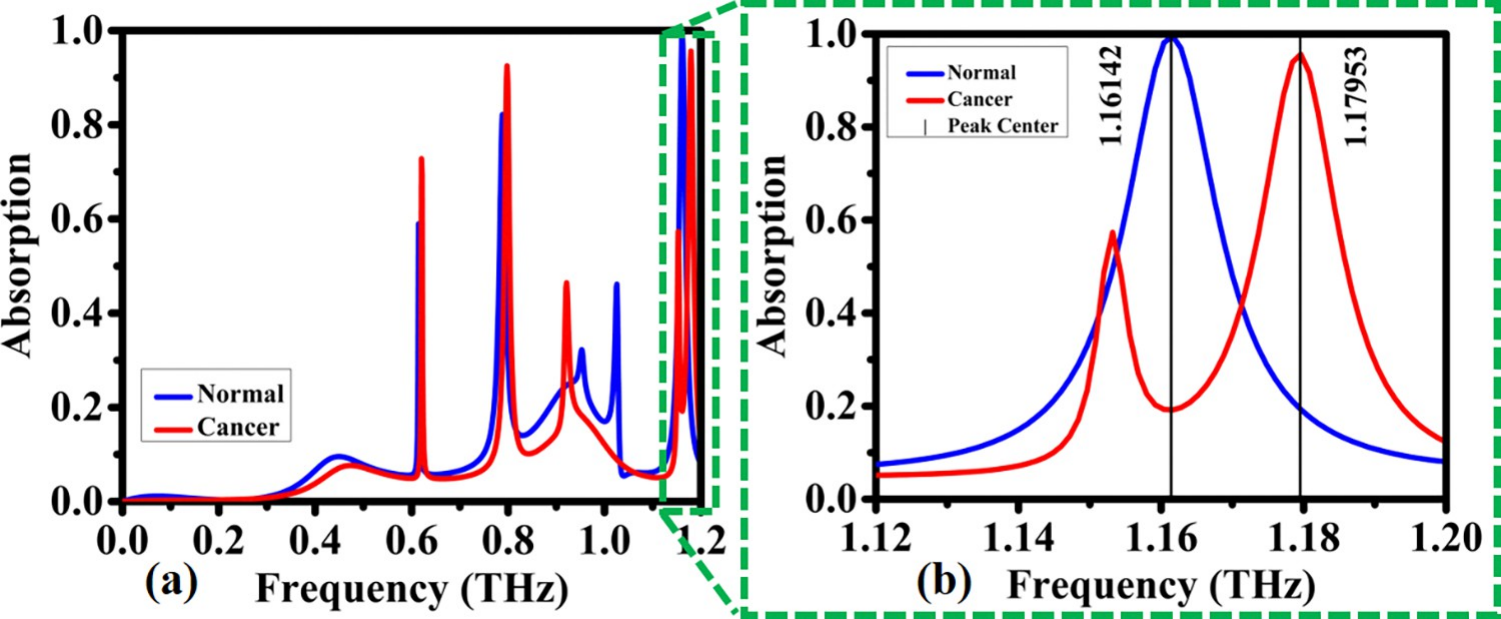
Detection of absorption coefficients by the proposed biosensor for normal blood and blood cancer within the frequency range of: (a) 0–1.2 THz, (b) 1.12–1.20 THz.

This biosensor utilizes cutting-edge technology to detect the presence of cancer cells in the human body at an early stage. By accurately identifying cancer cells, this biosensor can help in the early diagnosis and treatment of cancer, potentially saving lives. The Third Band Cancer Biosensor is a game-changer in the field of cancer detection, offering hope for improved outcomes for patients battling this disease. So as can be seen in Fig 28, third band is evaluated. Similar to previous case, blood cancer has blue shift from normal blood. This case leads to perfect absorption and occurred on 1.8 THz (see Fig 28B).

**Fig 29:**
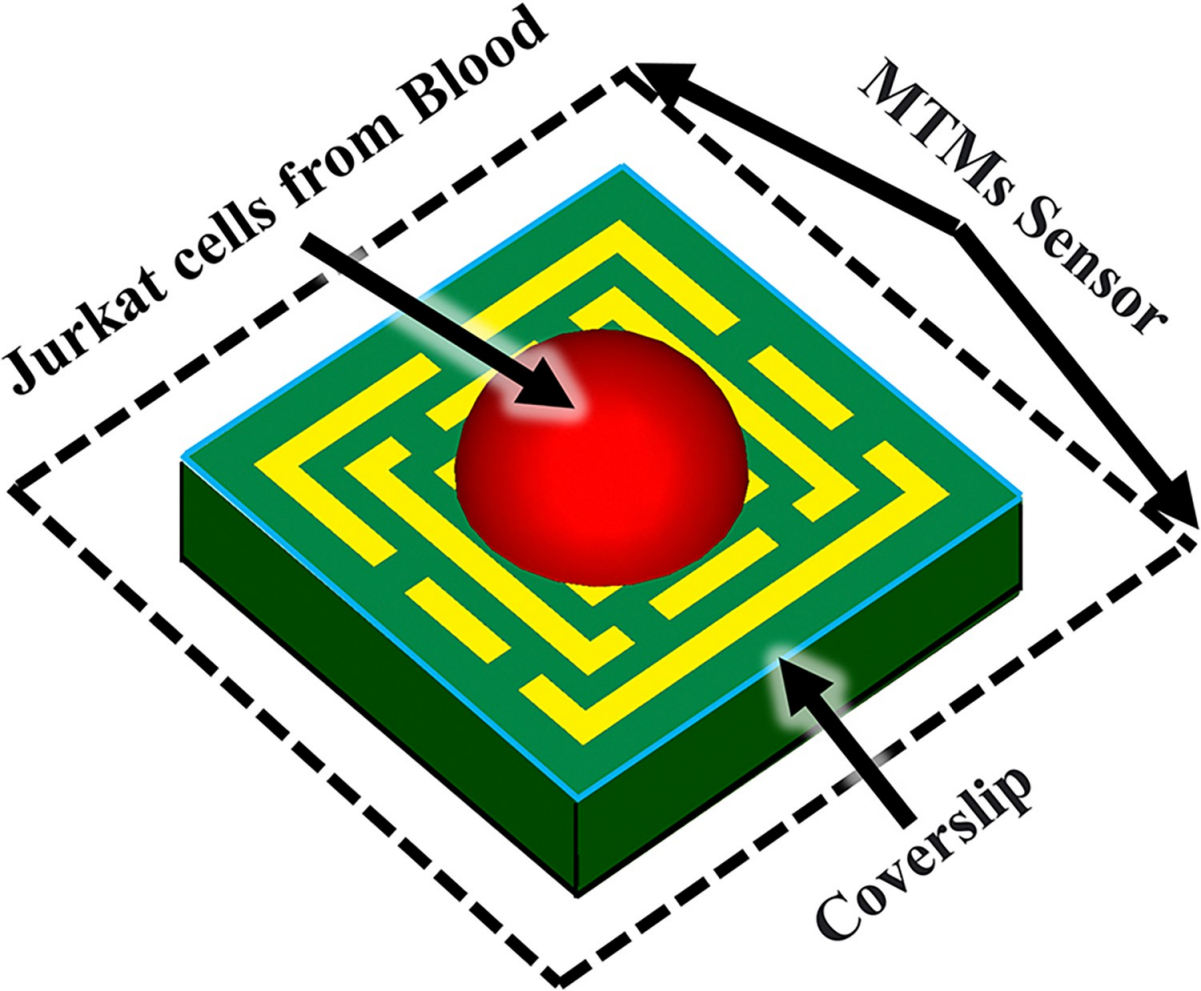
Utilization of the MWI approach for diagnosing blood cancer.

In order to further investigate the structure, MWI method is considered in Fig 29. The MWI (Magnetic Wire Interference) technique for blood cancer biosensors is an innovative approach that utilizes magnetic nanoparticles functionalized with antibodies specific to blood cancer biomarkers. These nanoparticles are then introduced to a blood sample, where they bind to the cancer biomarkers, creating a magnetic signal that can be detected by a sensor. This technique offers high sensitivity and specificity, making it a promising tool for early detection and monitoring of blood cancer. The integration of MWI technology in biosensors could revolutionize the diagnosis and treatment of blood cancer, potentially improving patient outcomes.

**Fig 30:**
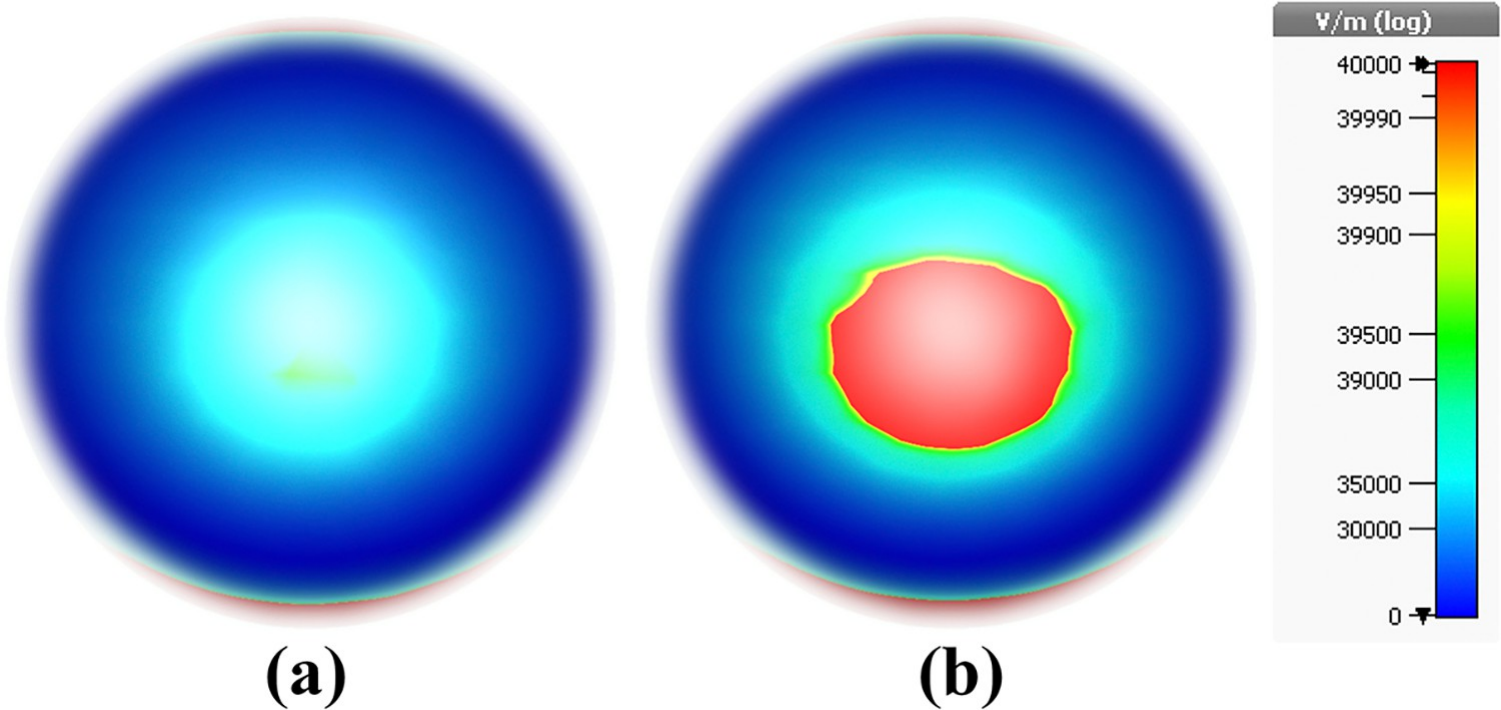
E-field results of the MWI technique: (a) Normal blood, and (b) Blood cancer.

**Fig 31:**
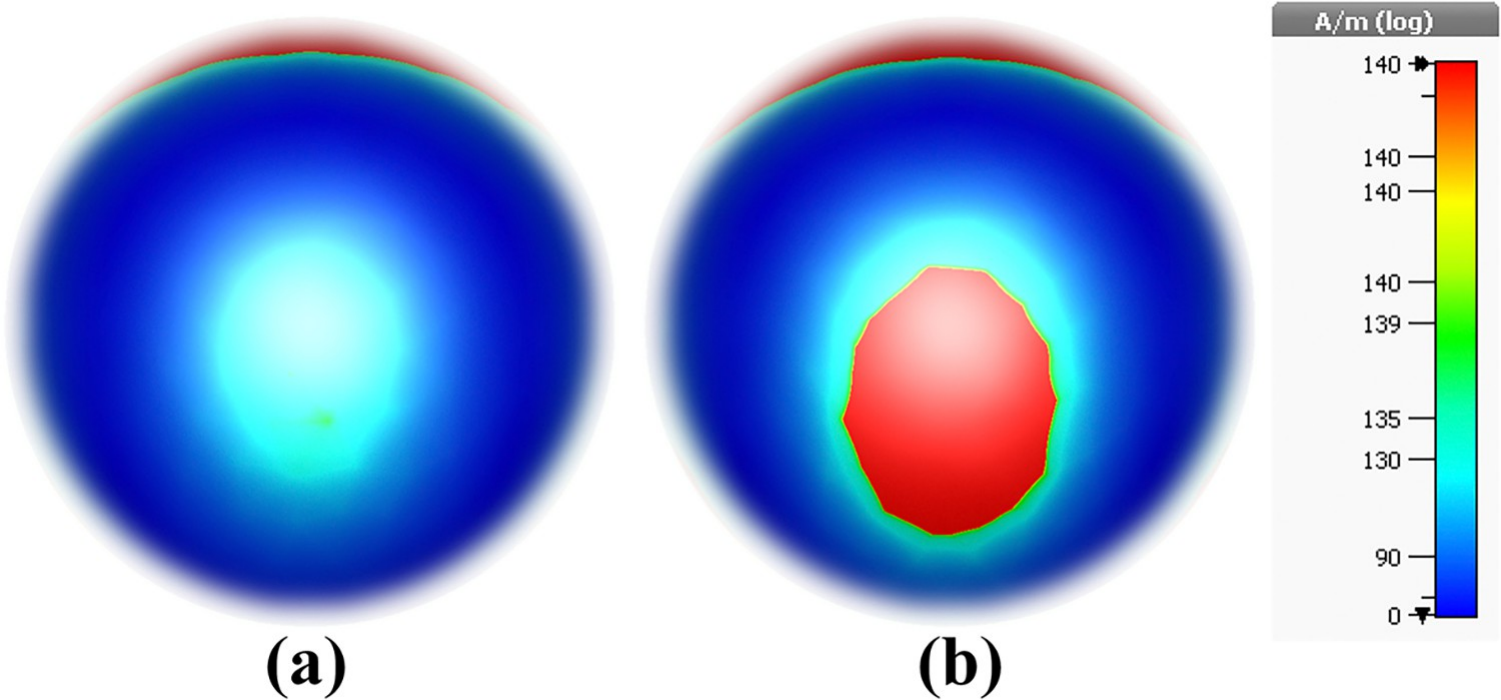
H-field results of the MWI technique: (a) Normal blood, and (b) Blood cancer.

Here, E-field profile is calculated for both normal and cancer cases. When cancer blood injected to structure, higher light-matter interaction is met. So, as can be seen in Fig 30, in case Fig 31A, E-field value is 3500 V/m and in case Fig 31B E-field value is 4000 V/m.

To verify the abovementioned results from E-field, here, MWI technique is applied for H-field. In detail, H-field, or magnetic field intensity, results in the MWI technique are used to map the distribution of electromagnetic properties within a target object. By examining changes in the H-field, researchers can identify variations in material composition and detect potential abnormalities, such as tumors in medical imaging applications. The MWI technique relies on the principles of electromagnetic wave propagation and reflection to create detailed images based on the interaction of microwave signals with tissues or other materials. It is a valuable tool in various fields, including medical diagnostics, non-destructive testing, and security screening.

As can be seen in Fig 31, higher light-matter interaction is occurred in blood cancer ones. In case Fig 31A, H-field value is 130 A/m and in case Fig 31B H-field value is 140 A/m.

The proposed THz MTM-based biosensor represents a significant advancement in the field of real-time blood cancer detection. By integrating this innovative biosensor into MWI systems, we have demonstrated a substantial enhancement in the efficacy and resolution of blood cancer diagnosis.

The biosensor’s quintuple-band design and high absorption rate enable the simultaneous detection of multiple biomarkers, providing a more comprehensive assessment of blood cancer status. This capability, in conjunction with the biosensor’s compact size and real-time monitoring capabilities, offers a significant advantage over traditional diagnostic methods.

Furthermore, the biosensor’s ability to accurately distinguish between healthy and cancerous cells underscores its potential for early detection of blood cancer. Early detection is crucial for improving treatment outcomes and increasing survival rates. By integrating this biosensor into MWI systems, we have taken a significant step towards realizing the potential of real-time blood cancer monitoring.

## 6. Future perspective

Terahertz (THz) electromagnetic (EM) wave imaging biosensors present a promising avenue for the early-stage detection of various cancers, including colorectal, cervical, adrenal gland (PC-12), breast, and non-melanoma skin cancers. These biosensors offer the advantages of real-time, high-resolution imaging and accurate diagnosis through non-ionizing radiation. Ongoing advancements in THz technology, materials, and miniaturization are poised to further enhance diagnostic capabilities, potentially revolutionizing cancer detection. Future research directions include expanding the application of these biosensors to other types of cancer or diseases, leveraging their multi-band functionality for broader diagnostic use. Additionally, investigating methods to further miniaturize the device could enable its integration into wearable or implantable diagnostic devices. Another critical area for future research is optimizing the sensitivity and selectivity of the biosensor through advanced metamaterial designs or hybrid systems. Finally, incorporating machine learning techniques into the MWI framework could facilitate real-time, automated analysis of detected biomarkers, thereby improving diagnostic accuracy and enabling personalized medical treatment.

## 7. Benchmarking

This section assesses the proposed quintuple-band terahertz metamaterial biosensor’s performance, focusing on sensitivity, Figure of Merit (FOM), and Quality Factor (Q-Factor). These metrics highlight the biosensor’s precision in detecting refractive index changes for early blood cancer diagnostics. Its multi-band operation and high SNR improve diagnostic accuracy and reliability, making it a competitive tool for real-time, non-invasive biomedical applications.

### 7.1 Performance evaluation and sensitivity metrics

The proposed ultra-compact quintuple-band terahertz metamaterial biosensor demonstrates remarkable performance when benchmarked against existing biosensing technologies. One of its most notable achievements is its Figure of Merit (FOM), reaching values of 140.77, 50.688, and 198.461 RIU−1 (as demonstrated in Table 2) across different operational frequencies. These FOM values, which are directly related to the biosensor’s sensitivity and resolution, highlight its exceptional capability in detecting minute changes in the refractive index, making it highly suitable for early-stage blood cancer diagnostics. Furthermore, the sensor exhibits impressive Quality Factor (Q-Factor) values of 207.378, 60.57, and 183.846 (as presented in Table 2) at corresponding resonance frequencies, underscoring its efficiency in energy confinement and resonance stability. A higher Q-Factor translates to sharper resonance peaks and a lower susceptibility to noise, thus improving the signal-to-noise ratio (SNR) and contributing to more accurate cancer detection.

**Table 2:** Bio-sensing performance comparisons of various sensor applications based on metamaterial.

Ref.	Year Published	FOM (RIU−1)	Q	S (THz/RIU)	Bio-application
[77]	2014	0.1216	5.58	0.02432	detection of Penicillia
[78]	2017	-	-	0.0242, 0.02438	detection of Virus
[79]	2020	-	-	0. 960	Biosensor, Collagen
[80]	2020	1.88	6.6	0.285	sensor
[81]	2021	-	-	0. 2833	Polystyrene particle
[56]	2021	-	-	0.074	Cervical cancer
[57]	2022	3.86	13	0.207	Cancer detection
[83]	2022	0.166	-	1.06	detection of avian influenza virus
[104]	2022	-	-	0.068	Hepatocellular carcinoma
[82]	2022	1.81, 1.57	8.21, 6.05	0. 203	sensor
[105]	2023	-	11	0.278	Bovin serum albumin protein
[59]	2023	-	82	0.495	Cancer detection
[60]	2023	0.86, 1.15	12.8, 13.5	0.0515, 0.076	Non-Melanoma Skin Cancer Diagnostics
This work	-	140.77 50.688 198.461	207.378 60.57 183.846	0.435 0.7 1.29	Blood cancer Diagnostics

In terms of sensitivity, the biosensor achieves values of 0.435, 0.7, and 1.29 THz/RIU (as shown in Table 2), which reflects its superior responsiveness to refractive index variations caused by cancerous cell presence. These values are critical in enhancing the device’s real-time detection capabilities, as they allow for the precise identification of subtle biomolecular changes associated with early cancer stages. The biosensor’s quintuple-band design offers several distinct advantages over existing single-band, dual-band, or even triple-band biosensors, which typically lack the operational flexibility to detect multiple biomarkers simultaneously. By operating over five distinct resonance bands, the proposed sensor expands its diagnostic potential, allowing it to differentiate between multiple cancer-related biomarkers and improving the overall specificity and accuracy of blood cancer diagnostics.

Additionally, the biosensor’s compact form factor and high absorption rates (98.5%, 97.5%, 99.5%, 99.76%, and 85% (as illustrated in Table 3) across its five resonance peaks) make it highly compatible with portable and real-time diagnostic systems. The device’s nearly perfect absorption (>95%) across most of its operational bands ensures minimal signal loss, further enhancing the overall SNR and contributing to more reliable diagnostic outcomes.

**Table 3:** A comparative analysis of terahertz band metamaterial studies and the proposed biosensor design.

References	Frequency operating THz	Material substrate	Techniques used	Absorptivity	Application
[35]	1−2.2	dielectric Teflon	Au/dielectric Teflon/Au	0.99	Sensor
[86]	2−6	SiO2	graphene/Au/SiO2/Au	0.99	Refractive index sensor
[85]	1.5–1.7	silicon dioxide	Gold/silicon dioxide/ Gold	0.972, 0.991	biosensor for detecting coronaviruses
[58]	0.5–2.5	SiO2	SiO2/Graphene	-	Breast cancer detection
[89]	0–3	PET	PET/FSS/UV glue/ Graphene	0.99, 0.80, 0.95	Multifunctional Tunable Terahertz
[88]	1−3	dielectric layer	Au/dielectric layer/Au	0.99, 0.99	Sensor
[92]	0.5−4.5	Topas spacer	graphene/Topas/Au	0.99, 0.98, 0.99	Ultra-Broadband Absorber
[55]	0–0.37	Glass	Glass/InSb/MgF2/InSb	0.998	Colon Cancer Detection
[84]	7–9.5	SiO2	Au/SiO2/ Graphene	0.98	Multi-Frequency Broadband and Ultra-Broadband
[87]	1−3	photonic crystal plate	bulk Dirac semimetal/photonic crystal/Au	0.97, 0.98, 0.99	Narrowband perfect absorber
[90]	0.7–5	Teflon	Ion gel/Graphene/Teflon/Gold	>0.96	polarization-sensitive
This work	0−1.2	PET (Polyethylene Terephthalate)	Al/ PET /Al	0.985, 0.975, 0.995, 0.9976, 0.85	Blood cancer, Microwave Imaging and Biosensor

### 7.2 Signal-to-noise ratio and design advantages

The design of the biosensor with a high Q-Factor significantly improves the SNR by reducing energy dissipation at resonance frequencies, thereby ensuring that the signal strength is well-preserved while minimizing background noise. This is particularly important in multi-band systems like the proposed biosensor, where the simultaneous detection of multiple biomarkers could otherwise introduce additional noise. However, the high Q-Factor and multi-band configuration work synergistically to suppress such noise, leading to more accurate and reliable readings. Furthermore, the high FOM values corroborate the biosensor’s ability to distinguish cancerous cells with greater precision, thus improving diagnostic reliability even in early disease stages.

The innovative quintuple-band architecture not only enhances detection accuracy but also provides versatility in identifying various cancer types, a feature that single-band and dual-band biosensors cannot offer. These advancements are expected to contribute significantly to the future of real-time, non-invasive blood cancer diagnostics and position the proposed biosensor as a cutting-edge solution in the biomedical field.

The compact size, high absorption rates, exceptional sensitivity, and multi-band operation make this biosensor highly competitive compared to state-of-the-art devices, positioning it as a pioneering tool for enhanced blood cancer diagnostics. The combination of superior FOM, Q-Factor, and SNR optimization ensures that the proposed sensor delivers highly accurate and reliable results, paving the way for significant advancements in biomedical diagnostics.

## 8. Conclusions

This study focused on real-time monitoring of blood cancer using microwave imaging (MWI) techniques. To enhance the efficacy and resolution of blood cancer detection, a novel terahertz (THz) biosensor structure was developed and investigated within the context of MWI. The proposed structure features a polyethylene terephthalate (PET) dielectric layer and two aluminum (Al) layers, with a metamaterial cell implemented on the top layer to achieve a nearly perfect penta-band absorber operating within the 0.6 to 1.2 THz frequency range. The device’s architecture is meticulously designed to ensure an absorption rate exceeding 95% across all operating frequencies. Full-wave electromagnetic (EM) simulations were employed to evaluate the biosensor’s performance, including analyses of absorption properties, refractive index, and electric field distribution at various frequencies within the operating band. The device was integrated into an MWI system, and comprehensive numerical studies demonstrated its suitability for blood cancer diagnosis. These studies highlighted the device’s ability to detect abnormalities, particularly in distinguishing between healthy and cancerous cells. Furthermore, the proposed sensor was benchmarked against state-of-the-art biosensors reported in recent literature. Our findings indicate that the new structure offers competitive performance across all major performance indicators. Additionally, its compact size is a significant practical advantage for THz-range monitoring. Future research could expand this biosensor’s application to other diseases, enhance miniaturization for wearable or implantable use, and improve sensitivity and selectivity through advanced metamaterials. Incorporating machine learning in the MWI system could enable real-time biomarker analysis, boosting diagnostic accuracy and supporting personalized medical treatment.

## 9. Next steps

This research identifies several critical next steps for advancing the application of terahertz (THz) technology in blood cancer diagnostics. These steps must be systematically addressed to ensure scientific rigor and clinical relevance.

The first priority is the fabrication of the proposed THz biosensor, which is designed to detect biomarkers associated with blood cancer. This fabrication process is essential to ensure the sensor achieves the necessary specifications for sensitivity, accuracy, and biological sample compatibility. After fabrication, the sensor must undergo rigorous validation using high-precision measurement equipment. This validation will confirm that the sensor operates within its intended frequency range and meets required sensitivity levels and performance standards, thereby confirming its readiness for clinical application.

Following validation, the sensor’s performance will be tested using a dry setup phantom. This setup simulates the interaction between THz waves and blood cancer biomarkers, allowing the refinement of the sensor’s performance in a controlled environment prior to testing with actual biological samples. This step is critical to ensure the sensor’s reliability and measurement accuracy in real-world clinical conditions.

Subsequently, approval from relevant ethical and medical regulatory bodies must be obtained to proceed with testing on human samples. This step ensures that all human trials adhere to strict ethical guidelines and medical regulations. Only after securing these approvals can the sensor be tested with real blood samples. These tests will involve a comprehensive series of measurements aimed at validating the sensor’s diagnostic capabilities in clinical conditions.

Once the data from real blood samples are collected, a detailed statistical analysis will be conducted. This analysis will assess the diagnostic accuracy of the sensor by measuring sensitivity, specificity, and predictive value. These metrics will provide key insights into the sensor’s effectiveness in detecting blood cancer. A clinical trial will then be required to evaluate the sensor’s performance in real-world medical settings, determining its feasibility as a diagnostic tool.

Several challenges are anticipated in this process, particularly in gaining medical acceptance of the THz sensor as a reliable diagnostic method. Additionally, obtaining regulatory approvals may be time-consuming due to the stringent protocols involved in introducing new medical technologies. Another significant challenge is the complexity of conducting THz measurements, which requires specialized knowledge and equipment. However, the development of indirect measurement techniques may offer a solution by making the THz measurement process more cost-effective and accessible for broader clinical use.
